# TGF‐β‐induced IGFBP‐3 is a key paracrine factor from activated pericytes that promotes colorectal cancer cell migration and invasion

**DOI:** 10.1002/1878-0261.12779

**Published:** 2020-09-01

**Authors:** Rocío Navarro, Antonio Tapia‐Galisteo, Laura Martín‐García, Carlos Tarín, Cesáreo Corbacho, Gonzalo Gómez‐López, Esther Sánchez‐Tirado, Susana Campuzano, Araceli González‐Cortés, Paloma Yáñez‐Sedeño, Marta Compte, Luis Álvarez‐Vallina, Laura Sanz

**Affiliations:** ^1^ Molecular Immunology Unit Biomedical Research Institute Puerta de Hierro‐Segovia de Arana Madrid Spain; ^2^ Bioinformatics Unit Biomedical Research Institute Puerta de Hierro‐Segovia de Arana Madrid Spain; ^3^ Basic Medical Sciences Department Faculty of Medicine Universidad San Pablo CEU Madrid Spain; ^4^ Pathology Department Hospital Universitario Puerta de Hierro Majadahonda Madrid Spain; ^5^ Bioinformatics Unit Spanish National Cancer Research Centre (CNIO) Madrid Spain; ^6^ Department of Analytical Chemistry Faculty of Chemistry Universidad Complutense de Madrid (UCM) Madrid Spain; ^7^ Immunotherapy and Cell Engineering Laboratory Department of Engineering Aarhus University Aarhus Denmark; ^8^ Cancer Immunotherapy Unit (UNICA) Hospital Universitario 12 de Octubre Madrid Spain; ^9^ Immuno‐oncology and Immunotherapy Group Biomedical Research Institute 12 de Octubre Madrid Spain

**Keywords:** colorectal cancer, IGFBP‐3, pericyte, TGF‐β, tumor microenvironment

## Abstract

The crosstalk between cancer cells and the tumor microenvironment has been implicated in cancer progression and metastasis. Fibroblasts and immune cells are widely known to be attracted to and modified by cancer cells. However, the role of pericytes in the tumor microenvironment beyond endothelium stabilization is poorly understood. Here, we report that pericytes promoted colorectal cancer (CRC) cell proliferation, migration, invasion, stemness, and chemoresistance *in vitro*, as well as tumor growth in a xenograft CRC model. We demonstrate that coculture with human CRC cells induced broad transcriptomic changes in pericytes, mostly associated with TGF‐β receptor activation. The prognostic value of a TGF‐β response signature in pericytes was analyzed in CRC patient data sets. This signature was found to be a good predictor of CRC relapse. Moreover, in response to stimulation by CRC cells, pericytes expressed high levels of TGF‐β1, initiating an autocrine activation loop. Investigation of secreted mediators and underlying molecular mechanisms revealed that IGFBP‐3 is a key paracrine factor from activated pericytes affecting CRC cell migration and invasion. In summary, we demonstrate that the interplay between pericytes and CRC cells triggers a vicious cycle that stimulates pericyte cytokine secretion, in turn increasing CRC cell tumorigenic properties. Overall, we provide another example of how cancer cells can manipulate the tumor microenvironment.

## Graphical abstract

In this work, we show that TGF‐β produced by colorectal cancer cells activates pericytes that in turn promote their tumorigenic properties through the release of soluble factors, including IGFBP‐3, with a prominent role in cancer cell migration and invasion. Moreover, co‐implantation of colorectal cancer cells and pericytes in immunodeficient mice increased tumor growth.

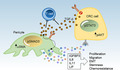

**Abbreviations**
CAF: cancer‐associated fibroblast
CRC: colorectal cancer
CSC: cancer stem cell
EC: endothelial cells
EMT: epithelial‐to‐mesenchymal transition
HSC: hepatic stellate cells
IGFBP‐3: insulin‐like growth factor‐binding protein‐3
PC: pericytes
TGF‐β: transforming growth factor‐β
TME: tumor microenvironment

## 1 Introduction

Pericytes (PC) are mural cells that embrace endothelial cells (EC) in capillaries, embedded in the same basement membrane [1]. The lack of specific markers and the heterogeneity of microvascular mural cells have been hurdles in PC identification [2], and only the advent of single‐cell RNA sequencing begins to allow the precise characterization of PC in normal tissues [3] and tumors [4]. PC have traditionally been credited with structural functions and trophic support to EC, being essential for vessel homeostasis. Accordingly, their functional role in the tumor microenvironment (TME) has been limited to vessel maturation during tumor angiogenesis. Indeed, PC are important for the maintenance of tumor neovasculature, and reduced PC coverage of tumor microvessels may impair vascular integrity and promote metastasis [5]. However, the role of PC in the TME is more complex, as they may contribute to different cancer hallmarks beyond tumor angiogenesis [6].

In the last years, EC have been implicated in the local supply of factors that might directly promote tumor growth in a paracrine fashion, independently from blood‐borne factors [7]. These ‘angiocrine’ factors, along with direct cellular contacts, could stimulate proliferation, promote the cancer stem cell (CSC) phenotype, and mediate tumor growth, metastasis, and resistance to chemotherapeutic agents [8]. For example, EC play an active role in promoting Notch signaling and the CSC phenotype through the NANOG pathway in colorectal cancer (CRC) [9]. EC‐initiated signaling can also enhance the survival, self‐renewal, and tumorigenic potential of primary human head and neck cancer stem‐like cells [10]. Closely related to these findings is the concept of perivascular niche where CSC finds refuge in the proximity of tumor vessels due to paracrine dependence on soluble factors secreted by EC [11]. Recently, it has been shown in a breast cancer model that disseminated tumor cells occupy the perivascular niche of distant tissues, where they are protected from chemotherapy [12]. This perivascular niche promotes important cues for cancer cell survival, stemness, and invasion [13].

Despite their privileged location in the interface between EC and tumor cells, the ‘nonvascular’ role of PC has been traditionally ignored and only a few recent studies address this issue [14]. In this line, it has been shown that PC conditioned media (CM) or PC coculture with thyroid carcinoma cells elicited resistance to vemurafenib and sorafenib, suggesting a protective effect [15]. Other works highlighted how PC promoted metastatic dissemination in a model of breast cancer [16] or contributed to establish the premetastatic niche in different target organs [17]. In a seminal study, PC were shown to promote ovarian cancer progression *in vivo* and predict poor prognosis in ovarian cancer patients [18], while fibroblasts did not affect tumor growth or metastasis. Still to be addressed is the identification of factors produced by PC that may contribute to these processes.

Here, we propose the CRC cell‐PC interplay as a new model to study epithelial–stromal interactions in the TME, and going a step further than previous works, we dissected at molecular level the bidirectional crosstalk between both cell types. Because cancer cells can instruct PC through major changes in their transcriptional profile and secretome, which in turn promote neoplastic cell proliferation, migration, stemness, and chemoresistance.

## 2 Materials and methods

### 2.1 Cells and reagents

Human brain vascular PC (ScienCell, Carlsbad, CA, USA), tested by ScienCell via immunofluorescence and PCR, and whose phenotypic characterization by FACS we have previously reported [19, 20], were cultured in PC medium (ScienCell). Human hepatic stellate cells (HSC) were also purchased from ScienCell and cultured in stellate cell medium (ScienCell). HCT116 (CCL‐247), HT‐29 (HTB‐38), and Caco‐2 (HTB‐37) CRC cell lines, as well as IMR‐90 human lung fibroblasts (CCL‐186) and CCD‐18Co fibroblasts from normal colon (CRL‐1459), were obtained from the American Type Culture Collection (ATCC, Manassas, VA, USA) and grown in DMEM (Lonza, Walkersville, MD, USA) supplemented with 10% FCS (Sigma‐Aldrich, St. Louis, MO, USA) and 1% pen‐strep‐glutamine (Gibco, Thermo Fisher Scientific, Waltham, MA, USA). PC and fibroblasts were used between passages 3 and 7. Cells were routinely screened for mycoplasma contamination by PCR (Biotools, Madrid, Spain) at the Tissue Culture Core Facility, Biomedical Research Institute Puerta de Hierro‐Segovia de Arana, and were authenticated at the Universidad Complutense de Madrid Genomics Unit using the AmpFLSTR Identifiler PCR Amplification Kit (Applied Biosystems, Thermo Fisher Scientific). Conditioned media (CM) were harvested after incubating 60% confluent cells for 72 h in DMEM 1% FCS, filtered through a 0.22‐μm filter, and stored at −20 °C.

The following reagents were used: TGF‐β1 (PeproTech, Rocky Hill, NJ, USA), IGFBP‐3 (PeproTech), EGF (PeproTech), MK‐2206 (Selleckchem, Houston, TX, USA), SB431542 (Sigma‐Aldrich), D‐luciferin (Promega, Madison, WI, USA), and anti‐TGF‐β antibody clone 1D11 (R&D Systems, Minneapolis, MN, USA). 5‐Fluorouracil (5‐FU) was obtained from the pharmacy at Hospital Puerta de Hierro.

### 2.2 Colorectal cancer cell proliferation

In cocultures with contact, equal numbers of PC or HSC and luciferase‐expressing HCT116 cells (HCT116^Luc^) cells were mixed in DMEM 1% FCS and plated in triplicates in 96‐well plates (2 × 10^3^/each/well). After 72 h, D‐luciferin (20 μg/well) was added and total light emitted was measured on the luminescence plate reader Infinite 1200 (Tecan, Männedorf, Switzerland). Transwell inserts for 24‐well plates with 0.4‐μm pore filters (Corning Life Sciences, Tewksbury, MA, USA) were used in cocultures without contact. HCT116, HT‐29, or Caco‐2 cells (3 × 10^4^) were plated in triplicates in 24‐well plates ON, whereas 10^4^ PC, HSC, CCD‐18Co, or IMR‐90 cells were seeded into transwell inserts. Then, media were replaced with DMEM 1% FCS and transwells were placed on top of the wells containing CRC cells. After 72 h, transwells were discarded and growth of CRC cells was assessed using CellTiter‐Glo Luminescent Cell Viability Assay (Promega).

### 2.3 Cell migration assays

In wound‐healing assays with HCT116, HT‐29, or Caco‐2 cells, Ibidi 2 well culture inserts for self‐insertion (Gräfelfing, Germany) with two reservoirs separated 500 μm were used. Inserts were adhered to the bottom of 24‐well plates, and 5 × 10^4^ cells were added into each reservoir. Simultaneously, 10^4^ PC were seeded into 0.4‐μm pore transwell inserts. The following day, culture inserts were removed, medium was changed to DMEM 1% FCS, and transwells with PC, HSC, or CCD‐18Co, or empty transwells, were placed on the top of migrating cells. Migration was monitored by taking sequential photographs of the gap, three images per well/time point, using an inverted microscope Eclipse TS100‐F (Nikon, Amstelveen, the Netherlands). The wounded area was quantified using imagej (https://imagej.nih.gov) and expressed as percentage of uncovered area.

### 2.4 Cell invasion assays

Invasive activity of HCT116 or HT‐29 cells was assessed using BioCoat Matrigel Invasion Chambers (Corning). CRC cells (2.5 × 10^4^ cells) were placed into the upper compartment of 24‐mm 8.0‐μm pore Matrigel‐coated transwell filters, whereas 8 × 10^4^ PC were seeded in 24‐well plates. The day after, media were replaced with DMEM 1% FCS and invasion chambers were placed in PC‐containing or empty wells. After 48 h, noninvasive cells were removed from the upper chamber with cotton swabs, filters were excised, and migrated cells were quantified using CellTiter‐Glo.

### 2.5 Aldefluor assay

The Aldefluor Kit (StemCell Technologies, Durham, NC, USA) was used following the manufacturer's instructions. Briefly, PC and HCT116 cells (5 × 10^5^ each one) were cultured alone in 60‐mm Petri dishes or cocultured for 48 h. Then, cells were trypsinized, suspended in Aldefluor assay buffer containing aldehyde dehydrogenase (ALDH) substrate, and incubated for 45 min at 37 °C. For each sample, an aliquot was incubated with the ALDH inhibitor diethylaminobenzaldehyde (DEAB) as a negative control. PC were stained with anti‐human PDGFRβ‐PE antibody (Clone PR7212; R&D Systems) and excluded from further analysis. Samples were acquired on a MACSQuant Analyzer (Miltenyi Biotec, Bergisch Gladbach, Germany) and quantified using FlowJo (BD Biosciences, Franklin Lakes, NJ, USA) at the Flow Cytometry Core Facility, Biomedical Research Institute Puerta de Hierro‐Segovia de Arana.

### 2.6 Colonosphere formation assay

HCT116 or HT‐29 cells were plated at a density of 3 × 10^3^ cells/well in 24‐well ultra‐low attachment plates (Corning). Cells were grown in standard sphere‐forming medium consisting of DMEM/F12 (Gibco) supplemented with 1×B27 serum‐free supplement (Gibco), 20 ng·mL^−1^ EGF (Gibco), and 20 ng·mL^−1^ bFGF (PeproTech). For coculture experiments, 0.4‐μm pore transwells with 10^4^ PC, HSC, or CCD‐18Co cells seeded the day before were placed on the top of the corresponding wells. After 5 days, colonospheres were measured using CellTiter‐Glo.

### 2.7 5‐fluorouracil cytotoxicity assay

To assess the effect of PC on HCT116 cell sensitivity to chemotherapy, HCT116 (3 × 10^4^ cells per well) were seeded in 24‐well plates and treated with 5 µm 5‐FU (IC_50_ for HCT116 cells) in monoculture or in coculture with 10^4^ PC in a transwell system. In another set of experiments, 5 µm 5‐FU was added to HCT116 cells forming colonospheres in low attachment plates as described above, in monoculture or in transwell coculture with 10^4^ PC. After 96 h, HCT116 cell viability was evaluated using CellTiter‐Glo.

### 2.8 Immunocytofluorescent staining

Cocultures of PC and HCT116 were stained after 48 h with anti‐PDGFRβ (Abcam, Cambridge, UK) and anti‐EpCAM (Clone Ber‐EP4; Dako‐Agilent, Glostrup, Denmark) antibodies. In another set of coculture experiments, pSMAD3 was stained in PC with the same antibody used in immunoblotting. HCT116 cells incubated with PC CM for 15 min were stained with anti‐EpCAM and anti‐pSTAT3 (Clone D3A7; Cell Signaling Technology, Danvers, MA, USA) antibodies. PC were cultured without contact with HCT116 cells for 48 h before staining with anti‐αSMA antibody (Clone 1A4; Dako‐Agilent). All cells were cultured onto Lab‐Tek chamber slides (Nunc, Roskilde, Denmark) or μ‐plates 24‐well black with clear coverslip bottom (Ibidi), fixed with 4% paraformaldehyde, permeabilized in 0.1% Triton X‐100 for 10 min, and incubated with the corresponding primary antibodies ON. Then, cells were incubated with anti‐mouse and/or anti‐rabbit Alexa Fluor 488‐, 546‐, or 647‐conjugated secondary antibodies (Invitrogen, Thermo Fisher Scientific) for 1 h. Finally, nuclei were stained with TO‐PRO‐3 (Invitrogen) in some experiments. Fluorescence images were captured with a confocal laser scanning microscope TCS SP5 (Leica Microsystems, Mannheim, Germany).

### 2.9 RNA extraction for microarray analysis

PC and HCT116 cells were mixed (5 × 10^5^ cells/each), seeded into 60‐mm Petri dishes, and incubated for 48 h. Monocultures were performed in parallel for the same lapse of time. Following coculture, cells were trypsinized and stained with anti‐human PDGFRβ PE‐conjugated mouse IgG_1_ antibody (R&D Systems) for fluorescent‐activated cell sorting into PDGFRβ+ and PDGFRβ− populations using a FACSAria II flow cytometry cell sorter (BD Biosciences). Total RNA was isolated using RNeasy Micro Kit (Qiagen, Hilden, Germany), quantified with a NanoDrop ND‐1000 spectrophotometer (Thermo Fisher Scientific), and checked for integrity on a Bioanalyzer 2100B (Agilent Technologies, Santa Clara, CA, USA). Three independent RNA samples were obtained for each condition, representing biological triplicates.

### 2.10 Global transcriptome profiling

Synthesis of cDNA, hybridization to Human Gene 2.0 ST arrays (Affymetrix, Santa Clara, CA, USA), and subsequent data processing were carried out at the Universidad Complutense de Madrid Genomics Unit as previously described [20]. The microarray data produced in this analysis were deposited in NCBI's Gene Expression Omnibus repository and are accessible through GEO Series Accession Number GSE108988.

### 2.11 Bioinformatic analysis

Heat maps were produced with the r program package version 3.0.2 (The R Foundation for Statistical Computing, Vienna, Austria; https://cran.r‐project.org/package=gplots) using the *ggplot2* and RColorBrewer libraries. The Database for Annotation, Visualization and Integrated Discovery (DAVID 6.7, http://david.abcc.ncifcrf.gov) bioinformatic tool for Gene Ontology was applied to determine the roles of these differentially expressed genes [21]. The gsea 3.0 software from the Broad Institute (https://www.gsea‐msigdb.org/gsea/index.jsp) was used to perform gene set enrichment analysis using the hallmark data set (one of the eight major MSigDB collections) [22].

A tumor‐associated PC gene signature with the genes differentially upregulated upon coculture with CRC cells was established without prior selection. To study the association of this signature with clinical parameters, we used the Affymetrix data set GSE14333 [23] publicly available in GEO with follow‐up information after surgery for 197 CRC patients. For this data set, it has been reported an association of CRC subtype with recurrence only in patients not receiving adjuvant treatment [24]. Therefore, this analysis was restricted to this group of patients in data set GSE14333. The Web‐based tool SignS (http://signs2.iib.uam.es/) [25] was used applying the threshold gradient descent method for the Cox model to build a predictive model for the risk based on the impact of the PC gene signature on the disease‐free survival (DFS). Kaplan–Meier DFS curves were generated for patients stratified in *quartiles* according to signature expression.

### 2.12 Quantitative real‐time PCR

To validate the gene expression profile determined by microarray analysis, a panel of selected genes were analyzed by quantitative real‐time PCR (qRT**–**PCR) in PC stimulated with 10 ng·mL^−1^ TGF‐β1 for 24 h. Total RNA was isolated with the RNeasy Mini Kit (Qiagen), and cDNA was derived from 500 ng of total RNA by random primed reverse transcription using NZY First‐Strand cDNA Synthesis Kit (Nzytech, Lisboa, Portugal). qRT**–**PCR was performed with a LightCycler 480 apparatus using the LightCycler 480 SYBR Green I Master Kit (Roche Diagnostics, Rotkreuz, Switzerland). Primer sequences (Table S1) were synthesized by Roche Diagnostics. Fold‐expression changes were calculated using the equation $2^{‐(\Delta \Delta C_{T})}$, as previously described. Each sample was tested in triplicate to account for intra‐assay variation.

### 2.13 Western blot

Pericytes were cultured alone with 10 ng·mL^−1^ TGF‐β1 or HCT116 CM for 1 h, or cocultured with HCT116 during 72 h. Cells were lysed in Laemmli lysis buffer (Bio‐Rad, Hercules, CA, USA) for 10 min on ice and collected by scraping. Equal amounts of proteins were resolved on 4–12% SDS/PAGE gels (Invitrogen) and transferred onto nitrocellulose membrane using iBlot Dry Blotting System (Invitrogen). Membranes were incubated ON with rabbit polyclonal antibody anti‐human p‐SMAD3 (#600‐401‐919; Rockland Immunochemicals, Pottstown, PA, USA) or anti‐human α‐SMA mouse monoclonal antibody (Clone 1A4; Abcam). HCT116 cells were incubated with 50 ng·mL^−1^ IGFBP‐3 for 15 min or 72 h, and cell lysates were assayed with rabbit anti‐human pAKT (Clone D9E) and anti‐human p42/44 MAPK (Clone D13.14.4E, both from Cell Signaling Technology) or mouse anti‐human N‐cadherin antibody (Clone D‐4; Santa Cruz Biotechnology, Dallas, TX, USA), respectively. Simultaneously, anti‐human β‐actin mouse monoclonal antibody (ab8226; Abcam), anti‐human β‐actin rabbit polyclonal (ab8227; Abcam), or anti‐human α‐tubulin mouse monoclonal antibody (Clone DM1A; Cell Signaling Technology) was added, diluted 1 : 2000, as a loading control. Primary antibodies were followed by 1 : 5000‐diluted DyLight 800‐conjugated anti‐rabbit or anti‐mouse antibody and DyLight 680‐conjugated anti‐rabbit or anti‐mouse antibody (Rockland Immunochemicals). Visualization and quantitative analysis of protein bands were carried out with the Odyssey Infrared Imaging System (LI‐COR Biosciences, Lincoln, NE, USA).

### 2.14 TGF‐β1 quantification

The use of an amperometric immunosensor for the sensitive determination of TGF‐β1 has been previously described [26]. The immunosensor implies the implementation of a sandwich immunoassay onto the surface of carboxylic acid‐functionalized magnetic microparticles (Dynabeads M‐270 Immunoassay; Invitrogen) and coupled with screen‐printed carbon electrodes (DropSens‐Metrohm, Oviedo, Spain). Recombinant human TGF‐β1 standard, mouse anti‐TGF‐β1 capture antibody, and biotinylated chicken anti‐human TGF‐β1 detection antibody used for sandwich immunocomplex formation were components of the human TGF‐β1 DuoSet ELISA Kit (R&D Systems).

### 2.15 Cytokine secretion profiling

PC (5 × 10^5^ cells) were seeded into 60‐mm Petri plates. After 24 h, the medium was replaced with DMEM 1% FCS with or without 10 ng·mL^−1^ TGF‐β1 and CM were collected after 48 h. The Proteome Profiler™ Human XL Cytokine Array Kit (#ARY022B; R&D Systems) was used to measure semiquantitatively the changes in 105 cytokines and chemokines in culture supernatants according to manufacturer's instructions. Instead of the HRP‐conjugated streptavidin provided with the kit, 10 ng·mL^−1^ IRDye 800‐labeled streptavidin (Rockland Immunochemicals) was used for detection with the Odyssey imaging system.

### 2.16 Lentiviral vectors and cell transduction

A third‐generation lentiviral vector packaging system was used along with the transfer vector pRRL‐Luc‐IRES‐EGFP driving the expression of firefly luciferase and EGFP [27]. Lentiviral particles were produced by cotransfection of 293T cells through the calcium phosphate precipitation method. HCT116 cells (1 × 10^5^) were seeded in 6‐well plates and transduced ON with lentiviral stocks at a final multiplicity of infection of 10. After 16 h, medium was replaced, and cells were cultured for 48 h. EGFP transgene expression was monitored by flow cytometry. For cell tracking, PC were infected with lentiviral particles encoding EGFP using the same protocol. Transduction did not affect cell viability and proliferation as assessed using CellTiter‐Glo.

### 2.17 Mice

Female athymic nude mice (Hsd: Athymic Nude‐*Foxn1^nu^*) 6–8 weeks of age were used for all experiments. Mice were obtained from Envigo (Huntingdon, UK) and housed under specific pathogen‐free conditions. All experiments were conducted in compliance with the institutional guidelines provided by the Puerta de Hierro‐Segovia de Arana Institute for Medical Research Animal Ethics Committee. Procedures were additionally approved by the Animal Welfare Division of the Environmental Affairs Council, Community of Madrid (PROEX 066/14).

### 2.18 *In vivo* tumor formation assays

HCT116 cells (500 cells) alone or mixed with 3 × 10^3^ PC in DMEM 30% Matrigel basement membrane matrix (BD Biosciences) were inoculated subcutaneously (s.c.) into nude mice. Tumors were measured every 3 days using a caliper, and volumes were determined using the formula width^2^ × length × 0.52. Mice were sacrificed when tumors reached 1 cm^3^ volume or became ulcerated. To assess the effect of PC on tumor implantation, HCT116^Luc^ cells were used in the same experimental setting and animals were imaged from day 4 to day 16 after inoculation.

### 2.19 *In vivo* bioluminescence imaging

Mice bearing HCT116^LUC^ or HCT116^LUC^ + PC xenografts were imaged using the high‐resolution charge‐coupled device cooled digital camera ORCA‐2BT (Hamamatsu, Hamamatsu City, Japan) as previously described [28]. Animals were injected intraperitoneally (i.p.) with 125 mg·kg^−1^ D‐luciferin 10 min before imaging and anesthetized using isoflurane (2.5% in 100% oxygen at a flow rate of 2 L·min^−1^ for induction and 1.5% and 1 L·min^−1^ during maintenance). Bioluminescence was collected with 2‐min integration time, and pseudocolor representations of light intensity were superimposed over the grayscale reference image acquired at low light. For quantification of the detected light, regions of interest were drawn and the light emitted from each region was assessed by recording total flux and maximal photon emission after background subtraction using the hokawo software (Hamamatsu).

### 2.20 *Ex vivo* EGFP tracking

Mice were co‐inoculated with HCT116 and PC^EGFP^ in the same conditions used previously. At days 14 and 30 after implantation, tumor xenografts were resected, embedded in Tissue‐Tek OCT compound, frozen in liquid nitrogen, and stored at −80 °C. 5‐μm sections were cut using a cryostat (Thermo Fisher Scientific), and fluorescence images were captured with a confocal laser scanning microscope TCS SP5 (Leica Microsystems).

### 2.21 Immunohistochemistry

Mouse CRC xenografts were routinely formalin‐fixed and paraffin‐embedded in the Department of Pathology, Hospital Universitario Puerta de Hierro. Sections of 4 µm were stained with hematoxylin and eosin according to standard protocols or processed for immunohistochemistry using the Dako‐Omnis automated staining platform. Primary antibodies anti‐Ki‐67 (Clone MIB‐1; Dako‐Agilent), α‐SMA (Clone 1A4; Dako‐Agilent), p‐STAT3 (Clone D3A7; Cell Signaling Technology), pAKT (Clone D9E; Cell Signaling Technology), ALDH (Clone 44/ALDH; BD Biosciences), and vimentin (Clone V9; Dako‐Agilent) were developed using the EnVision Flex, Low and High pH visualization system.

### 2.22 Statistical analysis

Results were expressed as mean ± standard deviation (SD). Data were evaluated using paired two‐tailed Student's *t*‐test and were considered to be statistically significant when *P* < 0.05. Asterisks used to indicate significance correspond with: *P < 0.05; **P < 0.01; ***P < 0.001. All statistical comparisons, data processing, and presentation were performed using prism software v7 (GraphPad, San Diego, CA, USA).

## 3 Results

### 3.1 Effect of pericyte coculture on the proliferation, migration, and invasion of colorectal cancer cells

**Fig. 1:**
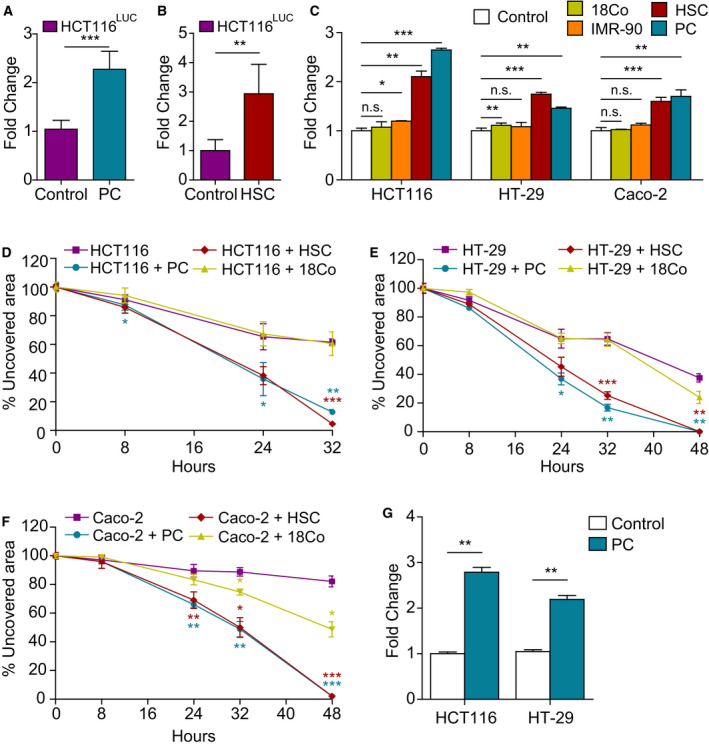
Coculture with pericytes promotes colorectal cancer cell proliferation, migration, and invasion. HCT116^Luc^ proliferation was quantified by bioluminescence after 72 h in coculture with contact with PC (A) or HSC (B) (*n* = 3). (C) HCT116, HT‐29, and Caco‐2 proliferation in monoculture or in coculture without contact with PC, HSC, CCD‐18Co, or IMR‐90 seeded on 0.4‐μm pore semipermeable transwells (*n* = 3). Wound‐healing assay with HCT116 (D), HT‐29 (E), or Caco‐2 cells (F) migrating in the presence of PC, HSC, or CCD‐18Co cells seeded on semipermeable transwells (*n* = 3). (G) Quantification of HCT116 and HT‐29 invasion through Matrigel‐coated 8‐μm transwells with PC growing in the bottom chamber (*n* = 3). Statistical differences were examined by paired Student's *t*‐test. Error bars indicate standard deviation. *P < 0.05; **P < 0.01; ***P < 0.001.

Initially, we tested whether juxtacrine or paracrine signaling between human primary PC and the human CRC cell line HCT116 could occur under different *in vitro* coculture setups, with and without direct cell**–**cell contacts. To this end, equal numbers of HCT116^Luc^ and PC were mixed and cultured for 72 h. HCT116^Luc^ cells cocultured with PC displayed a statistically significant increase in their growth rate (*P* < 0.0001), as assessed by bioluminescence quantification (Fig. 1A). This effect was not restricted to brain PC, since coculture of HCT116^Luc^ with liver PC (hepatic stellate cells, HSC) also promoted CRC cell growth (*P* = 0.0048) (Fig. 1B). To address whether this effect was dependent on direct cell**–**cell contact, cocultures were prepared in transwell inserts, with HCT116 cells seeded in the lower chamber and PC added to the upper chamber, separated by a semipermeable 0.4‐μm pore size membrane. After 72 h of coculture without contact, HCT116 cells exhibited increased proliferation compared with HCT116 cells alone (*P* = 0.0001), suggesting the implication of soluble factors produced by PC (Fig. 1C). Enhanced proliferation rate was not specific of HCT116 cells, since coculture with PC in transwells also promoted significantly the growth of HT‐29 (*P* = 0.0095) and Caco‐2 cells (*P* = 0.0029) (Fig. 1C). This effect was also observed in CRC cells using HSC in coculture with HCT116 (*P* = 0.0010), HT‐29 (*P* = 0.0009), and Caco‐2 (*P* = 0.0004). In addition, we included CCD‐18Co colon fibroblasts and IMR‐90 lung fibroblasts in the experiment for comparison. Interestingly, the enhancement of CRC cell proliferation by fibroblasts, although statistically significant in some cases, was less pronounced than that of PC in this model (Fig. 1C).

In order to assess whether soluble factors secreted by PC could also modulate the migratory properties of CRC cells, we used a wound‐healing assay with PC, HSC, or CCD‐18Co cells seeded on the upper chamber of semipermeable transwells and migrating HCT116, HT‐29, or Caco‐2 cells in the bottom. As shown in Fig. 1D, HCT116 cells cocultured with PC or HSC migrated significantly faster than HCT116 in monoculture (*P* = 0.0022 and 0.0009, respectively) and closed nearly completely the gap in 32 h, whereas HCT116 alone still left around 60% of denuded area at this time point. The presence of CCD‐18Co in the coculture system did not increase HCT116 migration rate. The effect of PC and HSC on HCT116 was reproduced using HT‐29 (Fig. 1E) or Caco‐2 cells (Fig. 1F). Coculture with CCD‐18Co also increased the area covered by Caco‐2 cells in the wound‐healing assay, although to a lesser extent (*P* = 0.019).

Next, chemoinvasion was tested by adding HCT116 or HT‐29 cells on the top of 8‐μm pore Matrigel‐coated transwell filters. PC in the bottom chamber considerably increased the total number of invading HCT116 and HT‐29 cells over controls (*P* = 0.0031 and *P* = 0.0057, respectively) when compared to empty bottom chambers (Fig. 1G).

### 3.2 Coculture of colorectal cancer cells with pericytes promotes cancer stem cell phenotype and chemoresistance

**Fig. 2:**
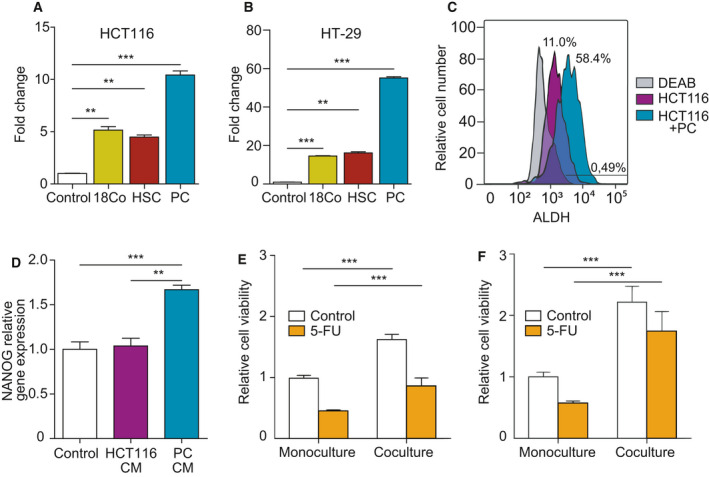
Coculture with pericytes enhances colorectal cancer cell CSC‐like phenotype. Formation of colonospheres by HCT116 (A) or HT‐29 cells (B) cultured alone or in coculture without contact with PC, HSC, or CCD‐18Co for 5 days (*n* = 3). (C) Coculture of HCT116 with PC cells increases ALDH1 expression, as assessed by flow cytometry. DEAB is an ALDH1 inhibitor used as negative control (*n* = 3). (D) *NANOG* relative gene expression in HCT116 colonospheres cultured in the presence of PC or HCT116 CM (*n* = 3). (E) Viability of HCT116 cells treated with 5 μm 5‐FU for 96 h in the absence or presence of PC in a transwell system (*n* = 3). (F) Protective effect of coculture with PC in HCT116 colonospheres treated with 5 μm 5‐FU (*n* = 3). Statistical analysis was performed using Student's *t*‐test. Error bars indicate standard deviation. **P < 0.01; ***P < 0.001.

In order to further understand CRC‐PC crosstalk, and especially the potential role of PC in promoting the stemness of CRC cells, a spheroid‐formation (colonosphere) assay was used. HCT116, HT‐29, or Caco‐2 cells were plated in low attachment wells and cultured in a serum‐free defined medium for 5 days, alone or in transwell coculture with PC, HSC, or CCD‐18Co. Caco‐2 cells did not form colonospheres under standard conditions of the assay and therefore were excluded. Coculture with HSC or CCD‐18Co significantly increased the sphere‐forming capacity of HCT116 (Fig. 2A) when compared with cells in monoculture (*P* = 0.0012 and *P* = 0.0020, respectively). However, this effect was more evident when PC were added to the upper chamber (*P* = 0.0005). Similarly, formation of colonospheres by HT‐29 cells was significantly increased in the presence of HSC or CCD‐18Co (*P* = 0.0015 and *P* = 0.0002, respectively), but especially when cultured with PC (*P* = 0.0001) (Fig. 2B). These results suggested that PC‐derived soluble factors could foster the CSC phenotype of CRC cells in a paracrine manner. As shown in Fig. 2C, the ALDH‐positive population presumably enriched for CSC also increased in the HCT116 cells cocultured for 48 h with PC from 11% to 58%, that is, a 5.3‐fold increase. PC CM also increased *NANOG* expression in HCT116 cells (*P* = 0.0002), a transcription factor essential for stemness which is expressed in multiple cancers, including CRC (Fig. 2D).

Cancer stem cell have been associated with increased resistance to chemotherapy and repopulation of tumors after treatment. In CRC, the most frequently used chemotherapeutic regimens (FOLFOX and FOLFIRI) contain 5‐FU. To interrogate the impact of PC on CRC chemoresistance, HCT116 cells were exposed to 5 μm 5‐FU in monoculture or in coculture with PC. After 96 h, viability of HCT116 cells in monoculture was 45% in comparison with untreated cells, while this percentage reached 56% in cocultured HCT116 cells (*P* < 0.0001) (Fig. 2E). This protective effect of PC was much more evident when 5‐FU was added to colonosphere‐forming HCT116 cells in low attachment plates (80% viable cells vs 57% in adherent HCT116 cells), consistent with an enrichment in chemoresistant CSC (*P* = 0.0002) (Fig. 2F).

### 3.3 Pericyte co‐implantation increases tumorigenicity of HCT116 cells *in vivo* and improves implantation rate

**Fig. 3:**
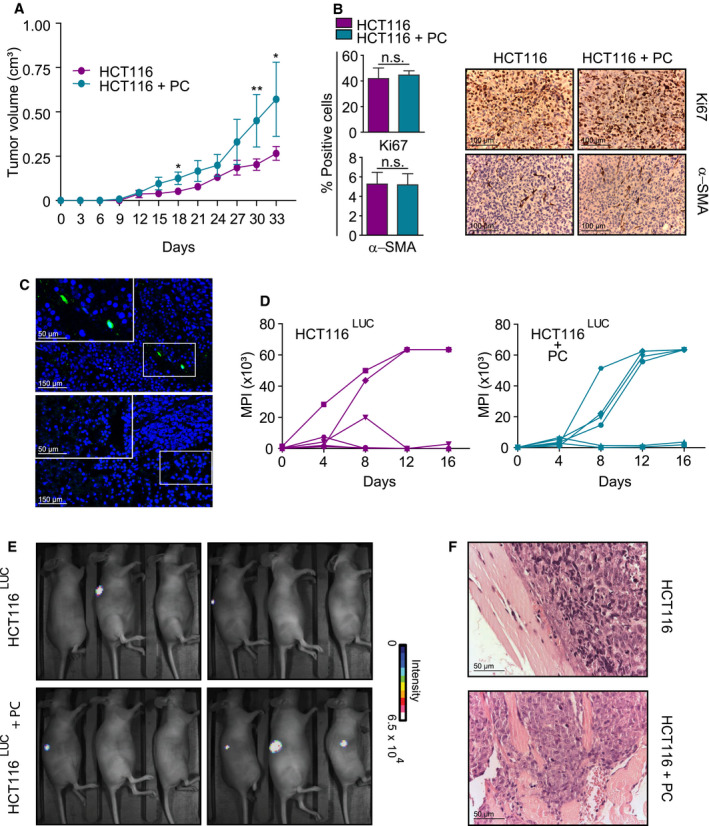
Pericytes promote *in vivo* tumor growth, implantation, and invasion. (A) Nude mice (*n* = 5/group) were inoculated s.c. with 500 HCT116 cells alone or co‐injected at a ratio of 1 : 6 with PC, and tumor volumes were measured every 3 days. (B) Quantification of Ki67‐ and αSMA‐positive cells in CRC xenografts resected at day 33 post‐inoculation. Data are shown as mean ± SD from three random fields from three tumors per group (left). Representative pathologic micrographs of Ki67 and αSMA immunostaining (right). Scale bar = 100 μm. (C) GFP‐engineered PC were tracked in HCT116 tumors at days 15 (top) and 30 (below) after co‐implantation. Representative images of two independent experiments. Scale bar = 150 μm (inset = 150 μm). (D) Implantation kinetics of tumors generated by HCT116^Luc^ alone (left) or co‐injected with PC (right) as assessed by bioluminescence imaging (*n* = 6/group). MPI: maximal pixel intensity of emitted light. (E) Bioluminescence images of nude mice (*n* = 6/group) co‐inoculated with HCT116^Luc^ plus PC and mice bearing HCT116^Luc^‐only tumors at day 16 post‐inoculation. Representative images of two independent experiments. (F) Representative H&E images of invasive edges at day 33 in HCT116 + PC tumors (top panel) compared with the smooth margins of controls (bottom panel) (*n* = 6/group). Scale bar = 50 μm. Statistical differences were examined by paired Student's *t*‐test. Error bars indicate standard deviation. ns, not significant; *P < 0.05; **P < 0.01.

The ability of PC to increase proliferation and support the CSC phenotype of HCT116 cells *in vitro* was suggestive of an increased tumorigenic potential *in vivo*. To address this issue, HCT116 cells were injected s.c. with or without PC (ratio 1 : 6) in the right flank of nude mice after 48 h of coculture in a transwell system. As shown in Fig. 3A, PC significantly augmented the growth rates of CRC xenografts compared to tumors with HCT116 alone (*P* = 0.005 at day 30 post‐inoculation and *P* = 0.02 at day 33). However, Ki67 staining of tumors resected at day 33 post‐inoculation revealed no differences in proliferation between both groups. Interestingly, the number of SMA+ cells at this time point was also similar (Fig. 3B). We had previously reported that endothelial cells in patient‐derived CRC xenografts were rapidly substituted by their murine counterparts [29]. As the anti‐SMA antibody used for immunohistochemistry is not species‐specific, we could speculate that most of the SMA+ cells detected at the end of the experiment came from the murine host. To corroborate this hypothesis, we analyzed the fate of co‐inoculated PC by transducing them with a lentiviral vector to constitutively express GFP. At day 15 post‐inoculation, we detected a small proportion of GFP‐positive cells in HCT116‐derived xenografts, which constituted less than 1% of all cells (Fig. 3C). Moreover, by day 30, no GFP‐expressing cell could be detected. Therefore, the advantageous effect of PC on HCT116 cells should take place at early time points, before human PC substitution. To assess this point, we injected s.c. HCT116^Luc^ cells alone or with PC (ratio 1 : 6) to follow the early fate of CRC cells by bioluminescence imaging before tumors were measurable. By day 16 post‐inoculation, 4 out of 6 tumors without PC showed no detectable photon emission. In contrast, 4 out of 6 tumors with HCT116 cells plus PC had reached top photon emission detected by the imaging system at that time point (Fig. 3D,E), suggesting a role for PC in tumor cell implantation. Remarkably, co‐inoculation of HCT116 cells with PC generated more frequently tumors that invaded into the muscular wall, as assessed by pathological analysis of the advancing edges (Fig. 3F).

### 3.4 Pericyte activation of TGF‐β signaling upon coculture with HCT116 cells

**Fig. 4:**
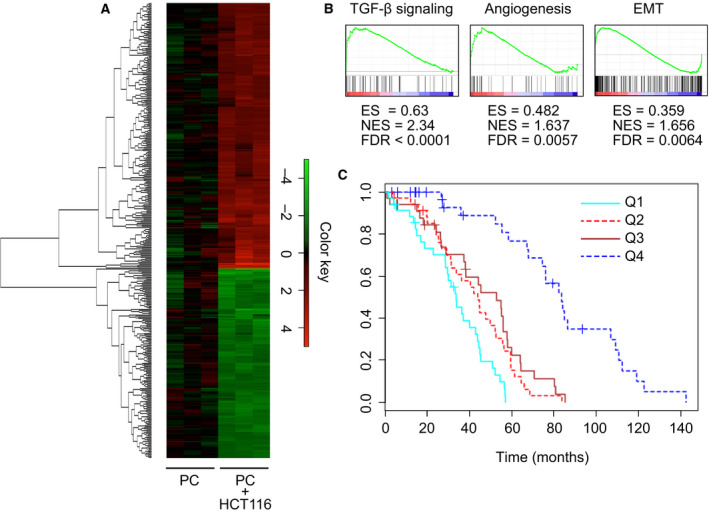
Gene profiling of cocultured pericytes by genome‐wide expression analysis. (A) Cluster heat map shows log2‐fold change values of differentially expressed genes (FDR < 0.05) in PC cocultured with HCT116 for 48 h vs PC in monoculture (*n* = 3). The complete list of genes used to generate the heat map is in Table S2. Results represent three independent experiments. (B) Gene set enrichment analysis (GSEA) of genes upregulated in cocultured PC. ES, enrichment score; NES, normalized enrichment score. (C) Kaplan–Meier curves for disease‐free survival (DFS) according to quartiles of the PC gene signature expression in patients with CRC from a publicly available external data set (GSE14333).

To investigate how HCT116 cells could modulate PC to increase their tumorigenic properties, the transcriptional profile of PC cocultured with HCT116 cells was analyzed. Equal numbers of cells were mixed and incubated for 48 h. Clumps of rounded HCT116 could be observed in light images, separated by tracts of elongated PC (Fig. S1A). The neat compartmentalization of each cell type was confirmed by confocal microscopy after staining of PDGFRβ (PC marker) and EpCAM (epithelial marker) (Fig. S1B). The cocultures were trypsinized and PDFGRβ+ cells were sorted, obtaining a PC population of > 99% purity (Fig. S1C). Total RNA from sorted PC and from control monoculture PC was isolated and used for microarray analysis (GEO Series accession number GSE108988). A total of 396 genes were significantly modulated [fold change > 2 or < 0.5, false discovery rate (FDR) < 0.05] in PC after coculture with HCT116 cells (Table S2). Heat map analysis of the transcriptome data allowed us to visually compare the levels of transcripts in each sample of the two groups (Fig. 4A). Gene ontology clustering analysis using DAVID confirmed the enrichment in the biological process (BP) category of the terms ‘blood vessel morphogenesis’, ‘vasculature development’, and ‘angiogenesis’. Interestingly, significant enrichments were also found for the BP term ‘transforming growth factor‐beta receptor signaling pathway’ and the molecular function (MF) term ‘SMAD binding’ (Table S3).

To further investigate the function of the upregulated genes in the PC molecular signature, we performed gene set enrichment analysis (GSEA) (Table S4). Out of the 50 hallmark gene sets, 10 gene sets met the threshold criteria (FDR ≤ 0.05), including angiogenesis and epithelial‐to‐mesenchymal transition (EMT) gene sets. Interestingly, only the TGF‐β signaling gene set reached a cut‐off value of normalized enrichment score (NES) > 2.0 (Fig. 4B). In fact, numerous genes were shared with the TGF‐β response signature previously described in different types of CRC stromal cells, including cancer‐associated fibroblasts (CAF) and EC [30] (Fig. S2A). Moreover, the expression of *TGFB1* was also significantly induced in cocultured PC, suggesting the onset of an autocrine regulatory loop in PC by TGF‐β produced by HCT116 cells. Of note, the PC gene signature was a good predictor of disease relapse in CRC patients and segregated a low‐expression group with significantly increased disease‐free survival after surgery (*P* < 1e‐7), providing a clinical correlate for our experimental findings (Fig. 4C). To verify that the gene expression profile determined by microarray analysis was indeed modulated by TGF‐β signaling, PC were treated with exogenous TGF‐β1 and the expression of selected genes from Fig. S2A (including *SNAI1*, *ZEB2,* and *VCAN*) was analyzed by qRT**–**PCR (Fig. S2B). In accordance with microarray data, upregulated genes in cocultured PC were also overexpressed by TGF‐β1‐treated PC.

**Fig. 5:**
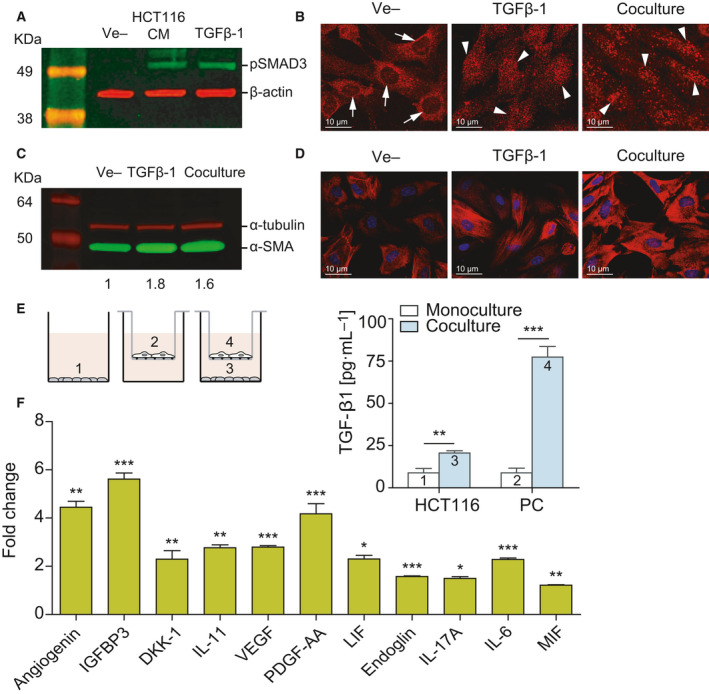
TGF‐β‐mediated crosstalk between pericytes and CRC cells modulates pericyte secretome. (A) Incubation in HCT116 CM for 1 h induces SMAD3 phosphorylation in PC, as assessed by western blot. Exogenous recombinant TGF‐β (10 ng·mL^−1^) was used as a positive control, and β‐actin was used as loading control (*n* = 3). (B) Confocal microscopy images of SMAD3 subcellular localization in PC cultured alone or cocultured with HCT116 cells for 48 h (*n* = 3). SMAD3 is detected in the cytoplasm of PC in monoculture (arrows show nonstained nuclei). Nuclear translocation of SMAD3 takes place after coculture with HCT116 cells for 48 h (arrowheads indicate stained nuclei). HCT116 cells treated with 10 ng·mL^−1^ TGF‐β1 were used as a positive control. Scale bar = 10 μm. (C) Western blot showing increased expression of αSMA in PC cocultured with HCT116 cells or stimulated with 10 ng·mL^−1^ TGF‐β1 for 48 h (*n* = 3). α‐tubulin was used as loading control. Numbers indicate the expression fold change relative to the loading control. (D) Representative confocal microscopy images of αSMA immunostaining of PC treated as in C. Nuclei were stained with TOPRO‐3 (blue). Scale bar = 10 μm. (E) TGF‐β1 quantification of the supernatants collected from top and bottom chambers of semipermeable transwells containing PC and HCT116, respectively, or each cell type cultured individually (*n* = 3). On the left, scheme of the coculture system used in this experiment. (F) Semiquantitative detection using antibody arrays of cytokines and chemokines in the CM of PC treated with 10 ng·mL^−1^ TGF‐β for 48 h (*n* = 3). Statistical analysis was performed using Student's *t*‐test. Error bars indicate standard deviation. *P < 0.05; **P < 0.01; ***P < 0.001.

To further assess that the TGF‐β pathway was activated in PC by HCT116‐produced TGF‐β1, we studied SMAD3 phosphorylation by western blot. As shown in Fig. 5A, HCT116 CM induced p‐SMAD3 as efficiently as exogenous TGF‐β1. Moreover, we demonstrated the nuclear translocation of p‐SMAD3 in PC cocultured with HCT116 cells (Fig. 5B). It has been reported that TGF‐β induced CAF transdifferentiation into αSMA myofibroblasts [31]. In this line, αSMA expression by PC increased in coculture with HCT116 cells or after TGF‐β1 treatment, suggestive of PC–myofibroblast transition. This was assessed by western blot (Fig. 5C) as well as by immunofluorescence (Fig. 5D).

In order to precisely dissect the role of TGF‐β in HCT116‐PC crosstalk, we quantified TGF‐β1 produced by HCT116 cells and PC in monoculture or cocultured according to the scheme shown in Fig. 5E. In monoculture, both cell types secreted similar amounts of TGF‐β1. However, in the coculture setting, TGF‐β1 production by PC showed a 8.7‐fold increase (*P* < 0.0001), confirming the existence of an autocrine loop (Fig. 5E). The apparent increase in TGF‐β secretion by CRC cells shown in lane 3 could be attributed to leakage from the upper chamber, as previously suggested in a similar experimental setting [32].

### 3.5 Secretome of TGF‐β‐treated pericytes

Data shown above suggested that HCT116 cells induced PC activation, which in turn provided HCT116 cells with increased tumorigenic properties, both *in vivo* and *in vitro*. Taking into account that most CRC cell lines have inactivated the TGF‐β pathway (including HCT116 cells), we addressed the impact of TGF‐β on PC secretome, since the effects of PC‐HCT116 crosstalk were contact‐independent. PC were treated with 10 ng·mL^−1^ TGF‐β1 for 48 h and supernatants were analyzed using antibody arrays (Fig. 5F), identifying a panel of eleven soluble factors whose secretion was significantly increased in comparison with untreated PC. As previously observed in cocultured PC with the DNA microarrays, an angiogenic program is launched by TGF‐β‐treated PC, including overexpression of VEGF, PDGF‐AA, and angiogenin. Among non‐angiogenic cytokines, IL‐6, IL‐11, and LIF have been reported to promote CSC‐like phenotype and chemoresistance mediated by STAT3 phosphorylation. Indeed, p‐STAT3 was detected in the nuclei of HCT116 cells upon incubation with PC CM (Fig. S3). Other soluble factors, such as IGFBP‐3 (the most overexpressed in TGF‐β‐treated PC, 5.6‐fold) and DKK1, have been hardly documented in this context. In order to address the clinical relevance of this panel in CRC, we interrogated The Pathology Atlas [33] for the association of each molecule with clinical outcome. According to this database, only *IGFBP3* out of the eleven secreted factors by TGF‐β‐treated PC was an unfavorable prognostic marker in CRC (www.proteinatlas.org/ENSG00000146674‐IGFBP3/pathology). The 5‐year survival in the low‐expression group (477 patients) was 65%, vs 46% in the 120 patients with high *IGFBP3* expression (*P* = 1.08e‐4).

**Fig. 6:**
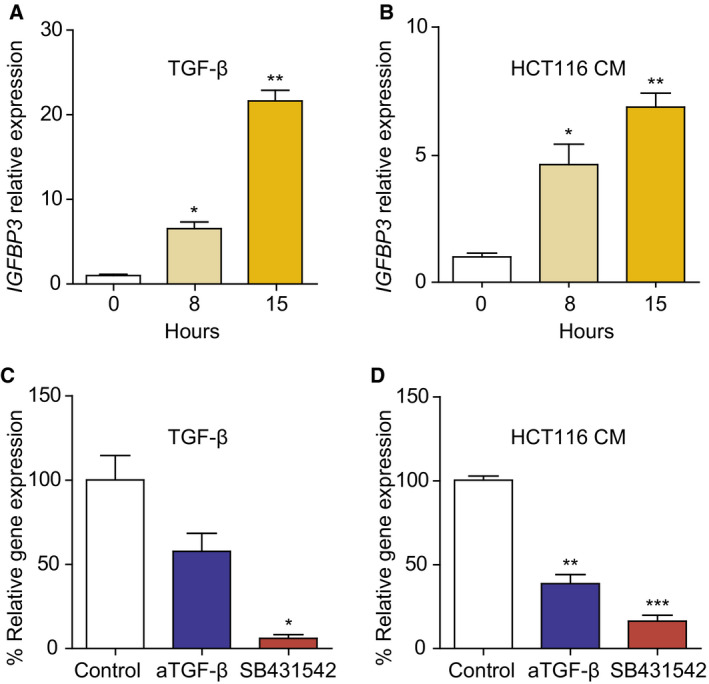
Transforming growth factor‐β1 induces insulin‐like growth factor‐binding protein 3 (IGFBP3) expression in human pericytes. *IGFBP3* mRNA expression, determined by qRT–PCR in PC exposed to DMEM 1% FCS (control), 10 ng·mL^−1^ TGF‐β (A), or HCT116 CM (B) for indicated time periods (*n* = 3). *IGFBP3* mRNA expression in PC treated with 10 ng·mL^−1^ TGF‐β1 (C) or HCT116 CM (D) in the absence or presence of 5 μg·mL^−1^ of pan‐TGF‐β‐neutralizing antibody 1D11 or 5 μg·mL^−1^ ALK5 antagonist SB431542 (*n* = 3). Relative expression of *IGFBP3* mRNA was normalized to that of *SDHA*. Each bar represents the mean ± SD of three independent experiments. Statistical analysis was performed using Student's *t*‐test. *P < 0.05; **P < 0.01; ***P < 0.001.

To further investigate the role of IGFBP‐3 in this context, first we confirmed by qRT–PCR that *IGFBP3* was upregulated in PC treated with recombinant TGF‐β1 (Fig. 6A) or HCT116 CM (Fig. 6B). Consistently, *IGFBP3* upregulation was substantially decreased by a neutralizing anti‐TGF‐β antibody and almost completely abrogated by the TGF‐β receptor inhibitor SB431542 in TGF‐β1‐treated PC (Fig. 6C) or PC cultured with HCT116 CM (Fig. 6D).

### 3.6 Functional effect of IGFBP‐3 on HCT116 cells

**Fig. 7:**
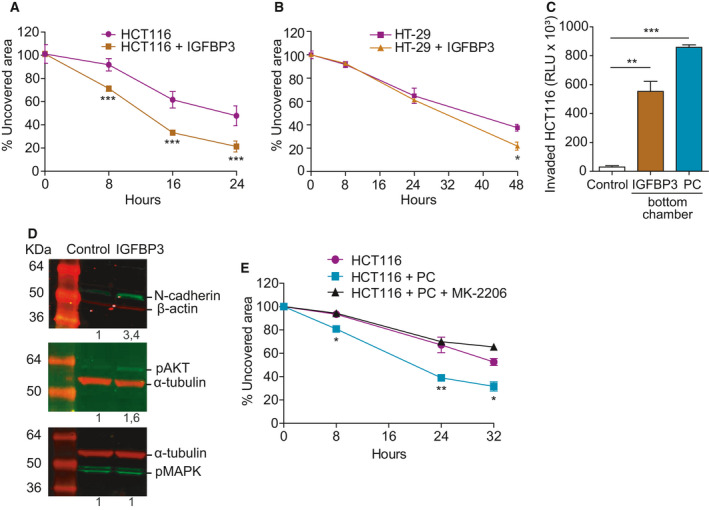
Insulin‐like growth factor‐binding protein 3 increases CRC cell migration and invasion through Akt activation. (A) HCT116 cell migration in the wound‐healing assay is promoted by 50 ng·mL^−1^ IGFBP‐3 (*n* = 3). (B) HT‐29 cell migration in the wound‐healing assay is promoted by 50 ng·mL^−1^ IGFBP‐3 (*n* = 3). (C) Chemoinvasion by HCT116 cells in Matrigel‐coated transwells significantly increases in the presence of 50 ng·mL^−1^ IGFBP‐3 (*n* = 3). Invasion toward PC is used as a positive control. (D) Treatment with 50 ng·mL^−1^ IGFBP‐3 for 72 h promotes the expression of N‐cadherin in HCT116 cells as assessed by western blot (top panel). Phosphorylation status of Akt (middle panel) and MAPK (bottom panel) in HCT116 cells treated with 50 ng·mL^−1^ IGFBP‐3 for 15 min. Representative images of three independent experiments (*n* = 3). Numbers indicate the expression fold change relative to the loading control. (E) Reversion of HCT116 cell migration promoted by PC with the AKT inhibitor MK‐2206 in wound‐healing assays (*n* = 3). Statistical analysis was performed using Student's *t*‐test. Error bars indicate standard deviation. *P < 0.05; **P < 0.01; ***P < 0.001.

Finally, we addressed the potential implication of IGFBP‐3 in the above described effects of PC on HCT116 cells. Treatment with recombinant IGFBP‐3 (50 ng·mL^−1^) significantly increased HCT116 cell proliferation, although at lower levels than those observed with PC coculture (Fig. S4A). Combination with EGF did not increase HCT116 growth rate, in contrast with previous reports on triple‐negative breast cancer cells. Moderate increases in HCT116 colonosphere formation (Fig. S4B) and chemoresistance to 5 μm 5‐FU (Fig. S4D) were observed, even though *NANOG* expression was higher in IGFBP‐3‐treated HCT116 colonospheres (Fig. S4C). On the other hand, IGFBP‐3 treatment had a profound impact on HCT116 migratory behavior. We initially examined whether IGFBP‐3 was able to modulate spontaneous HCT116 cell motility by supplementing IGFBP‐3 in culture in the wound‐healing assay. As shown in Fig. 7A, IGFBP‐3 significantly promoted HCT116 cell migration into the wounded area when compared to control cells (79% vs 52% of wound closure, respectively, at 24 h; *P* < 0.0001). This effect was also recapitulated by HT‐29 cells treated with IGFBP‐3, although to a lesser extent (Fig. 7B, *P* = 0.021). Similarly, Matrigel‐coated transwell invasion of IGFBP‐3‐treated HCT116 cells was considerably enhanced compared with that of untreated HCT116 cells (*P* = 0.0079) (Fig. 7C). It is widely accepted that increased invasiveness of cancer cell constitutes a hallmark of EMT. Indeed, IGFBP‐3 induced the upregulation of the mesenchymal marker N‐cadherin in HCT116 as assessed by western blot, in line with SGEA results (Fig. 7D).

Early studies had pointed to an important role for Akt in the regulation of multiple processes that control invasive migration. We found that AKT (but not MAPK) phosphorylation was induced in HCT116 cells upon IGFBP‐3 stimulation (Fig. 7D). In line with this observation, treatment with MK‐2206, a highly selective inhibitor of Akt1/2/3, inhibited PC‐induced migration of HCT116 cells (Fig. 7E). In tumor xenografts resected at day 33 post‐implantation, no differences in the expression of pAKT, pSTAT3, ALDH1, or vimentin could be detected in the HCT116 or the HCT116 + PC groups, consistent with an early effect of PC and their rapid substitution by host cells (Fig. S5).

## 4 Discussion

The relevance of PC as components of the TME and their contribution to malignant progression have traditionally been underestimated, due in part to the fact that the markers used to identify PC are frequently expressed in more widely recognized tumor stromal cells, and the widespread understanding that the abnormal tumor neovasculature lacks appreciable PC coverage. However, the presence of PC on tumor vessels may be ubiquitous, with coverage ranging from 50% to 95% in carcinomas and melanoma, although such PC may be more loosely attached to the vasculature in tumors than in normal tissues [34]. In fact, it has been shown that PC, once detached from tumor vessels, could be an important source of CAF/myofibroblasts as assessed by genetic tracing [35].

It has been known for decades that CAF are key players in the TME that promote tumor growth, invasiveness, and metastasis of many cancers. However, only recently has the concept of ‘cancer*‐*associated pericytes’ (CAP) been coined to designate FAP^+^ perivascular cells, which were phenotypically distinguishable from CAF by the absence of podoplanin [36]. Interestingly, both cell types exhibited similar expression of αSMA and TGF‐β, and their frequency was comparable in different murine tumor models such as LLC, B16, and MC38. Moreover, a seminal study using an ovarian cancer xenograft model demonstrated the key role of PC (here CD45^‐^ VLA‐1^bri^ cells) compared with CAF in promoting aggressive tumor growth *in vivo* [18].

In this work, we attempted to gain insight about the role of PC in the TME, using different CRC cell lines. HCT116 is a DNA mismatch repair‐deficient cell line resistant to TGF‐β due to biallelic mutational inactivation of *TGFBR2* [37] and has been assigned to the poor prognosis ‘mesenchymal’ CRC consensus molecular subtype (CMS) 4 [38]. CMS4 tumors have a significant overexpression of genes related to stromal infiltration, mesenchymal activation, and TGF‐β signaling [39]. Indeed, analysis of microdissected cells revealed that tumor‐associated stromal cells contributed mainly to the poor prognostic‐associated transcriptome compared with epithelial tumor cells [40].

Here, we show that PC‐cocultured CRC cells exhibited increased growth rate, migration, invasion, and stemness *in vitro*; moreover, co‐implantation of HCT116 plus PC promoted tumor growth *in vivo*. To gain knowledge on the molecular mechanisms implied, transcriptomic profiling of PC after coculture with HCT116 was performed. Not surprisingly, genes related to angiogenesis were significantly enriched, but a TGF‐β response signature was also prominent, in agreement with a previous study describing SMAD3 phosphorylation in different types of CRC stromal cells, whose TGF‐β activity promoted tumor initiation and increased metastasis [30]. Interestingly, the reciprocal interplay between PC and CRC cells also increased 8.7‐fold the release of TGF‐β by PC, suggesting an autocrine regulatory loop not previously described in PC.

Given that the enhancement of HCT116 protumorigenic properties appeared to be driven by soluble factors, we studied the secretome of TGF‐β‐stimulated PC and found 11 significantly upregulated proteins in the supernatant out of the 105 analyzed. It would be simplistic to blame a single factor for the variety of effects observed in CRC cells cocultured with PC. However, we can speculate that IL‐6, IL‐11, and LIF secreted by PC may be responsible for some of them, as they have well‐known roles in cancer. These cytokines activate the gp130‐JAK‐STAT3 pathway; in fact, we have demonstrated that PC CM triggers STAT3 signaling in HCT116 cells. In the work by Calon *et al*. [30], secretion of IL‐11 by TGF‐β‐stimulated CAF conferred a survival advantage to metastatic CRC cells. In addition, IL‐6 produced by CAF has been shown to support stemness of different CRC cell lines [41] and induce resistance to chemotherapy in gastric cancer [42]. Similarly, LIF overexpression has been reported to promote chemoresistance of HCT116 cells *in vitro* and *in vivo* [43].

Intriguingly, the soluble factor whose expression was most upregulated and the only one with prognostic significance in CRC patients, according to The Human Protein Atlas, was IGFBP‐3. As human PC have been shown to secrete IGFBP‐3 in response to IL‐1β [44], but there is no evidence in the literature of IGFBP‐3 production by PC in CRC, we focused on its functional role in this context. The insulin‐like growth factor (IGF)‐binding proteins (IGFBP) are a family of seven proteins that bind and transport IGF‐1 and IGF‐2, whose growth‐promoting effect can either inhibit or stimulate [45], and also have biological activity independent of IGF, as demonstrated using a mutant form of IGFBP‐3 [46]. Not surprisingly, the role of IGFBP family members in cancer is controversial: They were proposed as inhibitors, but elevated IGFBP‐3 serum levels predicted increased incidence of CRC [47] and high *IGFBP3* gene expression was associated with poor overall survival [48]. Moreover, secretion of IGFBP‐3 by tumor vs stromal cells exhibited contrasting effects on breast cancer progression [49]. *IGFBP3* upregulation in the tumor stroma of prostate cancers has been demonstrated by gene expression profiling following laser‐capture microdissection [50] and it has been suggested its role as mediator for tumor–stroma interactions [51]. In addition, IGFBP‐3 facilitated TGF‐β‐mediated EMT in esophageal [52] and lung cancer cells [53]. EMT has been classically associated with increased invasive activity of tumor cells, and indeed, IGFBP‐3 induced migration of tumor cells in nasopharyngeal carcinoma [54] and squamous cell carcinoma [46].

Here, we demonstrated that *IGFBP3* was overexpressed by PC incubated with exogenous TGF‐β or HCT116 CM. In line with previous works, we assessed that exogenous IGFBP‐3 or coculture with PC promoted migration and invasion of HCT116 cells, as well as N‐cadherin expression, consistent with the onset of an EMT program. Enhanced metastatic potential, EMT, and acquisition of CSC‐like traits are closely connected processes, and *NANOG* upregulation was another effect of IGFBP‐3 on CRC cells. Although the precise molecular mechanisms triggered by IGFBP‐3 remain to be elucidated, we have demonstrated AKT phosphorylation as part of the downstream signaling cascade.

On the other hand, the reciprocal interplay between PC and CRC cells not only enhanced tumorigenic properties of HCT116 cells, but also sustained the autocrine activation of PC by TGF‐β and their myofibroblastic differentiation, as evidenced by overexpression of αSMA, promoting a vicious circle to exacerbate tumor growth and invasion.

TGF‐β has also a well‐known role in supporting tumor immune escape and immunotherapy resistance [55]. Recently, it has been reported that increased TGF‐β promotes T‐cell exclusion in CRC [56] and it could be speculated that TGF‐β amplification by PC may act in synergy with tumor cells to inhibit local immune response. Furthermore, PC have a TGF‐β‐independent role in the inhibition of anti‐tumor immune responses, involving, among others, the expression of PD‐L1 and PD‐L2 and the secretion of nitric oxide and PGE2 [57, 58].

## 5 Conclusions

In summary, we propose here that PC, as multifaceted components of the TME, may contribute to a variety of cancer hallmarks, including not only tumor angiogenesis and immune suppression, but also increased tumor cell implantation and growth, invasion, and chemoresistance. Of note, both the PC gene signature and *IGFBP‐3* expression appear to identify CRC patients with poor prognosis. However, the role of PC is complex, as their removal may also increase metastasis and, consequently, their use as potential targets should be carefully evaluated. Understanding the complexity of the interplay between cancer cells and PC could offer additional insights into their functional contribution to cancer progression and response to therapy.

## Conflict of interest

The authors declare no conflict of interest.

## Author contributions

LS and RN designed the studies. RN, LM‐G, ES‐T, SC, AG‐C, and AT‐G performed experiments and statistical analysis and made the figures. CT and GG‐L performed bioinformatic analysis. CC provided histological and anatomo‐pathological studies. RN, PY‐S, MC, LA‐V, and LS analyzed and interpreted the data. LS and RN drafted the manuscript. All authors reviewed and agreed the final version of the article.

## Supporting information


**Fig. S1.** A, Cocultures of HCT116 cells and PC were maintained for 48 hours and evaluated with light microscopy (n = 3). Scale bar = 50 μm. B, Immunofluorescence staining of cocultures with antibodies against EpCAM (red) and PDGFRβ (green). Nuclei were stained with Topro‐3 (blue). Scale bar = 50 μm. C, Percentage of PDGFRβ positive cells (PC) in the coculture and after sorting (D). Three independent experiments were performed.

**Fig. S2.** A, Heat map of TGF‐β‐related genes in the PC signature, as reported by Calon *et al*. [30] in other stromal cells. B, Validation of DNA microarray data by qRT‐PCR in PC treated with 10 ng/ml TGFβ1 for 24h (n = 3). Error bars indicate standard deviation (SD).

**Fig. S3.** Confocal microscopy of pSTAT3 staining (red) in HCT116 treated with control medium (top) or PC CM (bottom) or. Nuclei were stained with Topro‐3 (blue) and EpCAM in green. Scale bar = 20 μm.

**Fig. S4.** Effect of IGFBP‐3 on HCT116 proliferation and stemness. A, Proliferation assay of HCT116 cells treated with 50 ng/ml IGFBP‐3, 50 ng/ml EGF, or their combination (n = 3). B, Colonosphere formation by HCT116 cells in the presence of 50 ng/ml IGFBP‐3 (n = 3). C, *NANOG* relative gene expression in HCT116 cultured in standard conditions, untreated HCT116 colonospheres and colonospheres stimulated with 50 ng/ml IGFBP‐3 for 5 days (n = 3). D, Viability of HCT116 cells treated with 5 μM 5‐FU in the presence or not of 50 ng/mL IGFBP‐3 (n = 3). Statistical analysis was performed using Student's t‐test. Error bars indicate standard deviation (SD). *, *P* < 0.05; ***, *P* < 0.001.

**Fig. S5.** Immunohistochemistry of vimentin, pAKT, pSTAT3 and ALDH1 in HCT116 or HCT116 + PC tumors resected at day 33 post‐inoculation. Scale bar = 50 μm.

**Table S1.** Primer sequences used in this study.

**Table S2.** Genes significantly modulated (fold change >2 or <0.5, FDR <0.05) in human primary pericytes cocultured with CRC cells. To avoid biases, we used the full set of genes without additional filtering.

**Table S3.** Gene ontology analysis of genes significantly upregulated (fold change >2, FDR < 0.05) from Table S2.

**Table S4.** GSEA analysis of gene sets significantly enriched (FDR < 0.05) in human primary pericytes cocultured with CRC cells.

## Data Availability

The microarray data produced in this work were deposited in NCBI's Gene Expression Omnibus repository and are accessible through GEO Series accession number GSE108988. Raw data are available from the corresponding author.
