# Supplementary figures and images for: TGF‐β‐induced IGFBP‐3 is a key paracrine factor from activated pericytes that promotes colorectal cancer cell migration and invasion
Source: Mol Oncol. 2020 Sep 1;14(10):2609–28. doi: 10.1002/1878-0261.12779 (PMC7530788; doi:10.1002/1878-0261.12779)

# Supplementary Figure 1

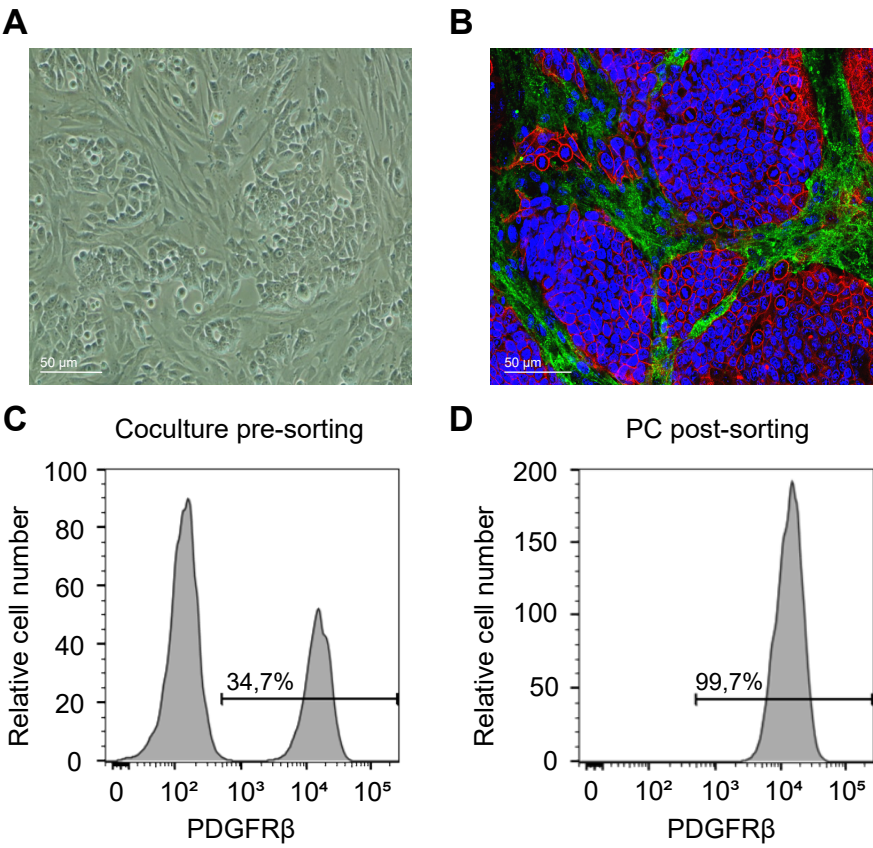

Supplement: Supplementary file 1 — Fig. S1. A, Cocultures of HCT116 cells and PC were maintained for 48 hours and evaluated with light microscopy (n = 3). Scale bar = 50 μm. B, Immunofluorescence staining of cocultures with antibodies against EpCAM (red) and PDGFRβ (green). Nuclei were stained with Topro‐3 (blue). Scale bar = 50 μm. C, Percentage of PDGFRβ positive cells (PC) in the coculture and after sorting (D). Three independent experiments were performed. [file MOL2-14-2609-s001.pdf]

# Supplementary Figure 2

A

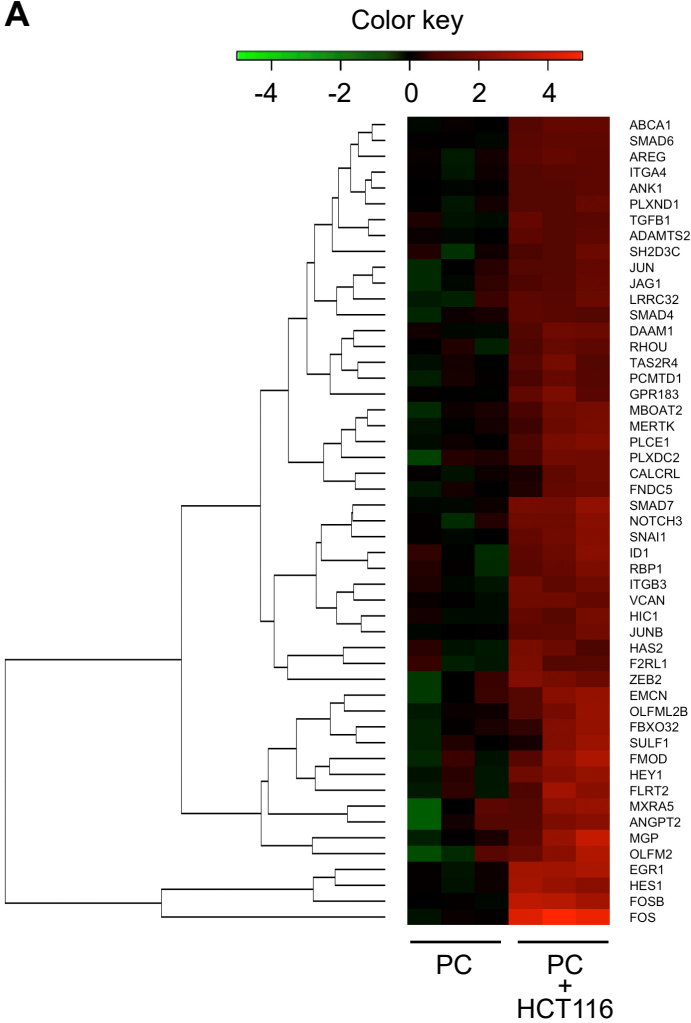

B

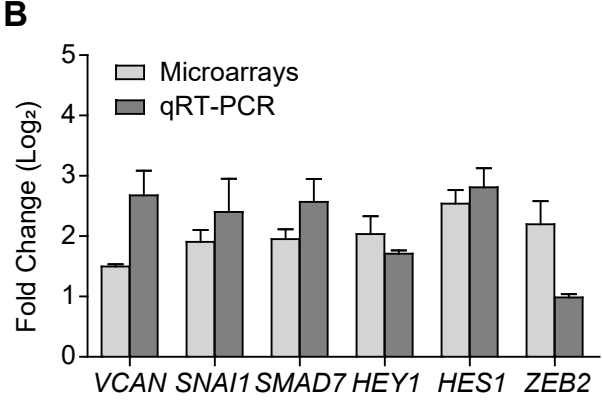

Supplement: Supplementary file 2 — Fig. S2. A, Heat map of TGF‐β‐related genes in the PC signature, as reported by Calon et al. [30] in other stromal cells. B, Validation of DNA microarray data by qRT‐PCR in PC treated with 10 ng/ml TGFβ1 for 24h (n = 3). Error bars indicate standard deviation (SD). [file MOL2-14-2609-s002.pdf]

## Supplementary Figure 3

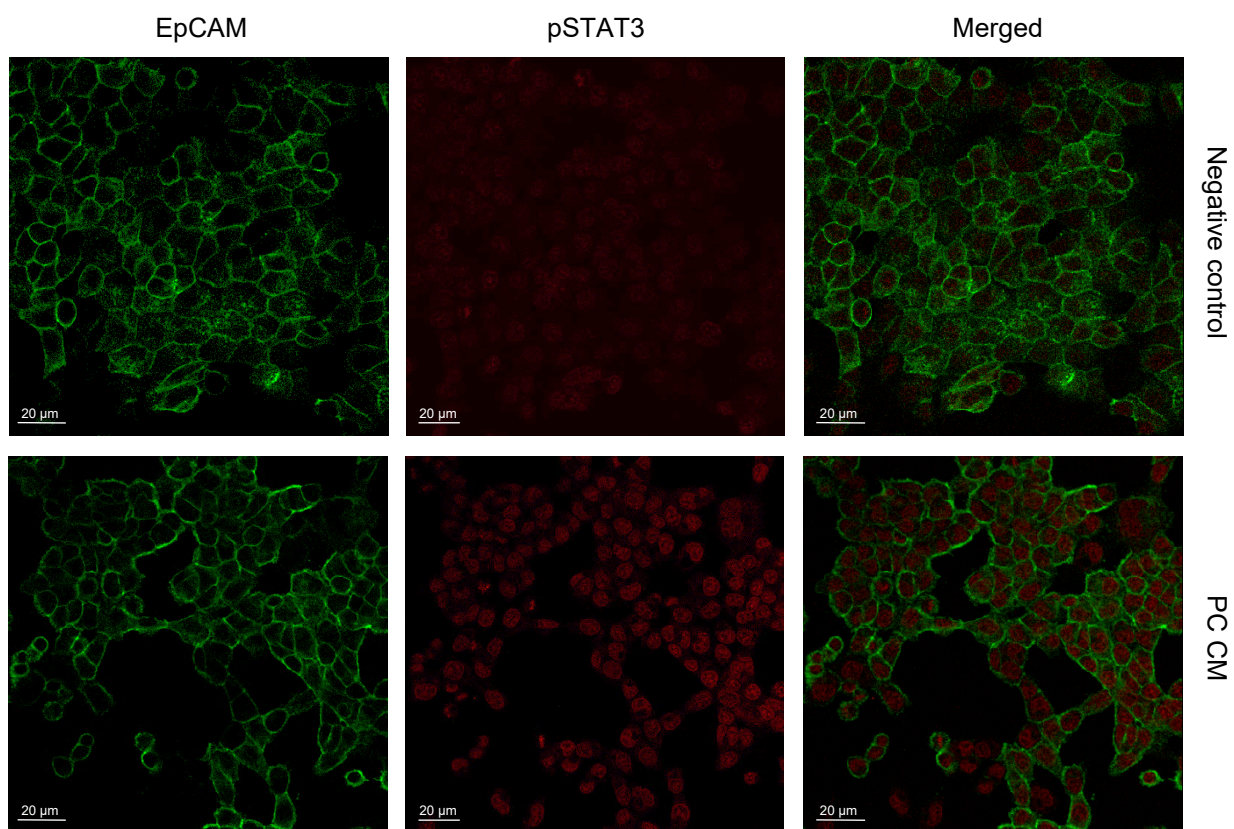

Supplement: Supplementary file 3 — Fig. S3. Confocal microscopy of pSTAT3 staining (red) in HCT116 treated with control medium (top) or PC CM (bottom) or. Nuclei were stained with Topro‐3 (blue) and EpCAM in green. Scale bar = 20 μm. [file MOL2-14-2609-s003.pdf]

# Supplementary Figure 4

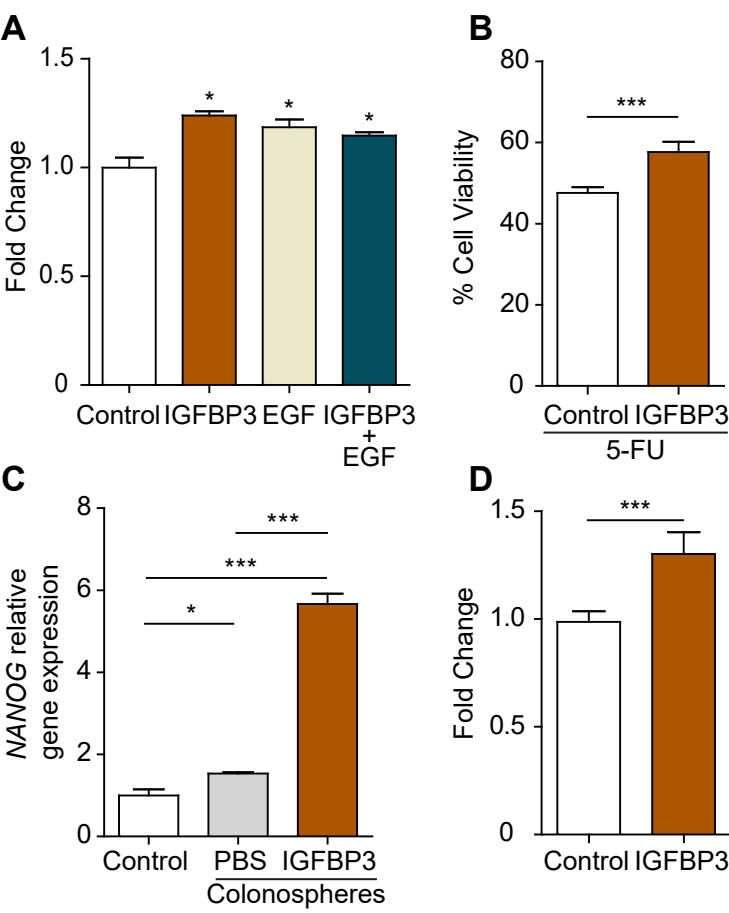

Supplement: Supplementary file 4 — Fig. S4. Effect of IGFBP‐3 on HCT116 proliferation and stemness. A, Proliferation assay of HCT116 cells treated with 50 ng/ml IGFBP‐3, 50 ng/ml EGF, or their combination (n = 3). B, Colonosphere formation by HCT116 cells in the presence of 50 ng/ml IGFBP‐3 (n = 3). C, NANOG relative gene expression in HCT116 cultured in standard conditions, untreated HCT116 colonospheres and colonospheres stimulated with 50 ng/ml IGFBP‐3 for 5 days (n = 3). D, Viability of HCT116 cells treated with 5 μM 5‐FU in the presence or not of 50 ng/mL IGFBP‐3 (n = 3). Statistical analysis was performed using Student's t‐test. Error bars indicate standard deviation (SD). *, P < 0.05; ***, P < 0.001. [file MOL2-14-2609-s004.pdf]

Supplementary Figure 5

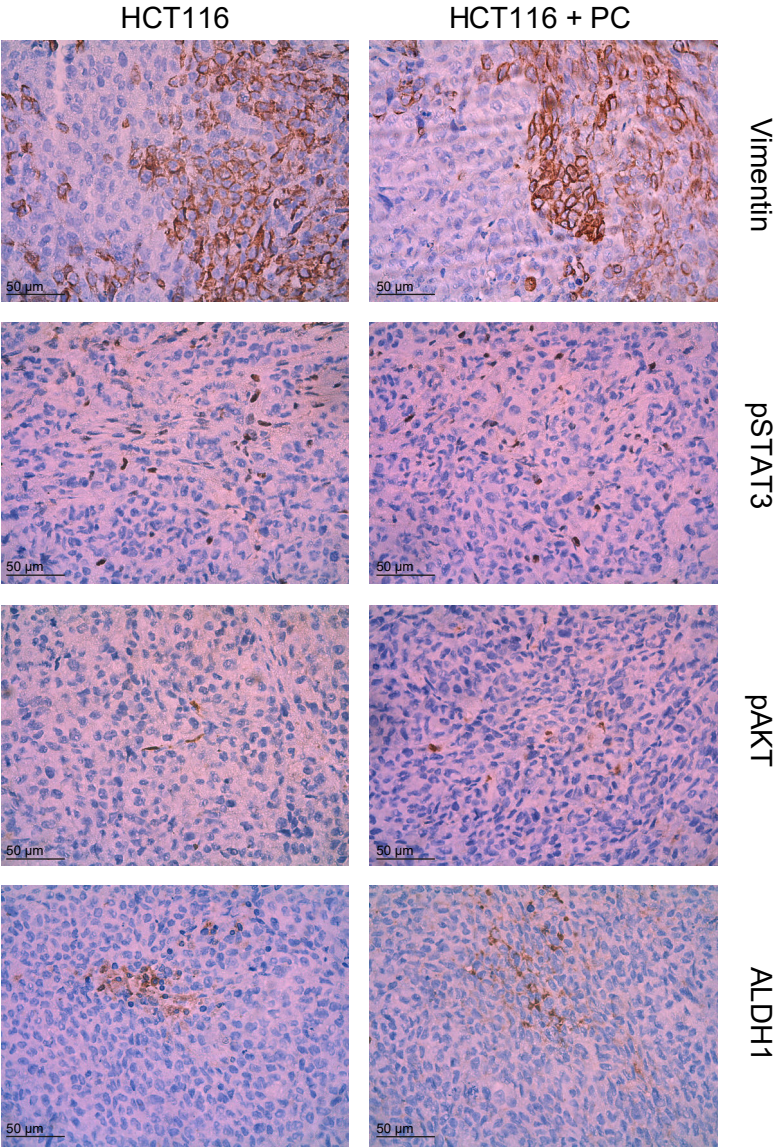

Supplement: Supplementary file 5 — Fig. S5. Immunohistochemistry of vimentin, pAKT, pSTAT3 and ALDH1 in HCT116 or HCT116 + PC tumors resected at day 33 post‐inoculation. Scale bar = 50 μm. [file MOL2-14-2609-s005.pdf]
