# Supplementary material for: TGF‐β‐induced IGFBP‐3 is a key paracrine factor from activated pericytes that promotes colorectal cancer cell migration and invasion
Source: Mol Oncol. 2020 Sep 1;14(10):2609–28. doi: 10.1002/1878-0261.12779 (PMC7530788; doi:10.1002/1878-0261.12779)
Supplement: Supplementary file 6 — Table S1. Primer sequences used in this study. [file MOL2-14-2609-s006.docx]

**Table S1.** Primer sequences used in this study.

| **Gene** | **Sequence (5’-3’)** | **Gene** | **Sequence (5’-3’)** |
| --- | --- | --- | --- |
| ***SDHA*** | (F) TGGGAACAAGAGGGCATCTG (R) CCACCACTGCATCAAATTCATG | ***HEY2*** | (F) GAGAAGACTTGTGCCAACTGCT (R) CCTGTTGCCTGAAGCATCTTC |
| ***SMAD7*** | (F) ACTGGTGCGTGGTGGCATA (R) AGCCATTCCCCTGAGGTAGAT | ***HEY1*** | (F) TCTGAGCTGAGAAGGCTGGT (R) AGGTGATCCACGGTCATCTG |
| ***SNAI1*** | (F) CACTATGCCGCGCTCTTTC (R) GGTCGTAGGGCTGCTGGAA | ***ZEB2*** | (F) TTTCCTGCCCTCTCTGTAG (R) GCTCCTTGGGTTAGCATTTGG |
| ***VCAN*** | (F) GTGTCACTGACTGTGGAT (R)CAAACAAGCCTTCTGAGC | ***IGFBP3*** | (F) AATCATCATCAAGAAAGGGC (R) GAACTTCAGGTGATTCAGTG |
| ***HES1*** | (F) GGAAATGACAGTGAAGCACCT (R) CAGCACACTTGGGTCTGTG | ***NANOG*** | (F)CCGACTGTAAAGAATCTTCACC  (R)GACAGAAATACCTCAGCCTCC |
