# Supplementary material for: TGF‐β‐induced IGFBP‐3 is a key paracrine factor from activated pericytes that promotes colorectal cancer cell migration and invasion
Source: Mol Oncol. 2020 Sep 1;14(10):2609–28. doi: 10.1002/1878-0261.12779 (PMC7530788; doi:10.1002/1878-0261.12779)
Supplement: Supplementary file 7 — Table S2. Genes significantly modulated (fold change >2 or <0.5, FDR <0.05) in human primary pericytes cocultured with CRC cells. To avoid biases, we used the full set of genes without additional filtering. [file MOL2-14-2609-s007.docx]

**Supplementary Table S2 -** Genes significantly modulated (fold change >2 or <0.5, FDR < 0.05) in human primary pericytes cocultured with CRC cells

| **Probe Set ID** | **Fold change** | **FDR** | **Gene Accession** | **Gene Symbol** | **Gene Description** | **mRNA Accession** |
| --- | --- | --- | --- | --- | --- | --- |
| **16786587** | 24,460 | 8,57E-08 | NM_005252 | FOS | FBJ murine osteosarcoma viral oncogene homolog | NM_005252 |
| **16971737** | 15,360 | 6,67E-06 | NM_000857 | GUCY1B3 | guanylate cyclase 1, soluble, beta 3 | NM_000857 |
| **16971712** | 14,421 | 1,11E-04 | NM_000856 | GUCY1A3 | guanylate cyclase 1, soluble, alpha 3 | NM_000856 |
| **16863287** | 9,271 | 7,72E-06 | NM_001114171 | FOSB | FBJ murine osteosarcoma viral oncogene homolog B | NM_001114171 |
| **16989736** | 7,186 | 3,18E-06 | NM_001964 | EGR1 | early growth response 1 | NM_001964 |
| **16761820** | 6,795 | 4,96E-03 | NM_000900 | MGP | matrix Gla protein | NM_000900 |
| **16920315** | 6,388 | 2,49E-03 | NM_001136021 | NFATC2 | nuclear factor of activated T-cells, cytoplasmic, calcineurin-dependent 2 | NM_001136021 |
| **16949759** | 5,898 | 1,75E-06 | NM_005524 | HES1 | hes family bHLH transcription factor 1 | NM_005524 |
| **16681840** | 5,293 | 3,60E-06 | --- | --- | --- | ENST00000408413 |
| **16912939** | 5,237 | 1,16E-03 | NR_030374 | MIR644A | microRNA 644a | NR_030374 |
| **17047985** | 5,020 | 2,41E-04 | NM_004194 | ADAM22 | ADAM metallopeptidase domain 22 | NM_004194 |
| **16698234** | 4,938 | 4,72E-03 | NM_002023 | FMOD | fibromodulin | NM_002023 |
| **17090564** | 4,876 | 5,38E-06 | NR_002914 | SNORD62A | small nucleolar RNA, C/D box 62A | NR_002914 |
| **17090566** | 4,876 | 5,38E-06 | NR_002914 | SNORD62A | small nucleolar RNA, C/D box 62A | NR_002914 |
| **16844061** | 4,804 | 4,06E-04 | NM_020405 | PLXDC1 | plexin domain containing 1 | NM_020405 |
| **17084413** | 4,787 | 1,26E-03 | --- | --- | --- | ENST00000515939 |
| **16852979** | 4,766 | 4,06E-04 | --- | --- | --- | NONHSAT059748 |
| **16868481** | 4,705 | 8,47E-03 | NM_001304347 | OLFM2 | olfactomedin 2 | NM_001304347 |
| **17012304** | 4,688 | 1,25E-03 | NM_012259 | HEY2 | hes-related family bHLH transcription factor with YRPW motif 2 | NM_012259 |
| **16946280** | 4,659 | 1,24E-02 | NM_022131 | CLSTN2 | calsyntenin 2 | NM_022131 |
| **16722212** | 4,618 | 7,29E-03 | --- | --- | --- | ENST00000528792 |
| **16964888** | 4,607 | 8,26E-07 | NM_001297559 | HTRA3 | HtrA serine peptidase 3 | NM_001297559 |
| **17005072** | 4,595 | 3,30E-04 | --- | --- | --- | ENST00000364497 |
| **17045538** | 4,546 | 8,46E-03 | --- | --- | --- | ENST00000517174 |
| **16762154** | 4,334 | 4,63E-02 | NM_005691 | ABCC9 | ATP binding cassette subfamily C member 9 | NM_005691 |
| **17047585** | 4,308 | 2,36E-04 | --- | --- | --- | NONHSAT121619 |
| **16787135** | 4,211 | 1,16E-02 | NM_013231 | FLRT2 | fibronectin leucine rich transmembrane protein 2 | NM_013231 |
| **17078452** | 4,148 | 1,43E-04 | NM_001040708 | HEY1 | hes-related family bHLH transcription factor with YRPW motif 1 | NM_001040708 |
| **16685704** | 3,968 | 2,58E-05 | NM_014571 | HEYL | hes-related family bHLH transcription factor with YRPW motif-like | NM_014571 |
| **16855127** | 3,958 | 2,35E-04 | NM_001190821 | SMAD7 | SMAD family member 7 | NM_001190821 |
| **16836624** | 3,930 | 1,09E-05 | NR_029493 | MIR21 | microRNA 21 | NR_029493 |
| **16996969** | 3,910 | 2,64E-02 | AK124130 | GUSBP3 | glucuronidase, beta pseudogene 3 | AK124130 |
| **17117545** | 3,896 | 3,04E-03 | AY358248 | LOC100131541 | uncharacterized LOC100131541 | AY358248 |
| **17065899** | 3,884 | 3,33E-04 | --- | --- | --- | NONHSAT124851 |
| **16960922** | 3,881 | 2,46E-04 | NM_002888 | RARRES1 | retinoic acid receptor responder (tazarotene induced) 1 | NM_002888 |
| **17069816** | 3,762 | 4,55E-02 | NM_001128204 | SULF1 | sulfatase 1 | NM_001128204 |
| **17118321** | 3,743 | 4,64E-04 | --- | --- | --- | ENST00000602979 |
| **16978354** | 3,739 | 9,74E-03 | NM_001159694 | EMCN | endomucin | NM_001159694 |
| **16812009** | 3,698 | 4,34E-03 | NR_026813 | LINC00597 | long intergenic non-protein coding RNA 597 | NR_026813 |
| **16984968** | 3,693 | 5,77E-04 | --- | --- | --- | NONHSAT101555 |
| **16919181** | 3,628 | 1,76E-04 | NM_001029864 | KIAA1755 | KIAA1755 | NM_001029864 |
| **16695741** | 3,616 | 3,96E-03 | NM_001297713 | OLFML2B | olfactomedin like 2B | NM_001297713 |
| **16712086** | 3,615 | 3,73E-02 | NM_001291494 | ITGA8 | integrin alpha 8 | NM_001291494 |
| **17080788** | 3,594 | 2,32E-02 | NM_001242463 | FBXO32 | F-box protein 32 | NM_001242463 |
| **17114697** | 3,572 | 1,12E-04 | --- | --- | --- | NONHSAT138810 |
| **16863534** | 3,564 | 4,07E-02 | --- | --- | --- | AK022126 |
| **16879476** | 3,549 | 1,60E-02 | --- | --- | --- | NONHSAT070343 |
| **17092115** | 3,517 | 4,37E-03 | XM_011517768 | GLIS3 | GLIS family zinc finger 3 | XM_011517768 |
| **16714086** | 3,444 | 2,05E-02 | NM_001031746 | VSTM4 | V-set and transmembrane domain containing 4 | NM_001031746 |
| **16928204** | 3,404 | 1,70E-03 | NR_003714 | POM121L9P | POM121 transmembrane nucleoporin-like 9, pseudogene | NR_003714 |
| **16979339** | 3,390 | 3,17E-03 | NM_001083 | PDE5A | phosphodiesterase 5A, cGMP-specific | NM_001083 |
| **17117888** | 3,387 | 5,77E-04 | ENST00000628473 | ZEB2 | zinc finger E-box binding homeobox 2 | ENST00000628473 |
| **17108816** | 3,386 | 4,01E-02 | NM_015419 | MXRA5 | matrix-remodelling associated 5 | NM_015419 |
| **16907641** | 3,375 | 3,48E-04 | --- | --- | --- | ENST00000428777 |
| **16869769** | 3,374 | 3,20E-04 | NM_000435 | NOTCH3 | notch 3 | NM_000435 |
| **16914791** | 3,308 | 1,01E-04 | NM_005985 | SNAI1 | snail family zinc finger 1 | NM_005985 |
| **16733038** | 3,287 | 6,22E-05 | --- | --- | --- | ENST00000410912 |
| **16681827** | 3,285 | 2,46E-02 | NM_004753 | DHRS3 | dehydrogenase/reductase (SDR family) member 3 | NM_004753 |
| **16784410** | 3,258 | 6,90E-03 | --- | --- | --- | BC020583 |
| **17118390** | 3,255 | 2,36E-04 | AK021795 | MGC24103 | uncharacterized MGC24103 | AK021795 |
| **16874267** | 3,244 | 1,40E-04 | XM_011526860 | SLC6A16 | solute carrier family 6, member 16 | XM_011526860 |
| **16699862** | 3,214 | 2,33E-03 | NR_039896 | MIR4742 | microRNA 4742 | NR_039896 |
| **16970118** | 3,186 | 2,56E-03 | NM_001128933 | SYNPO2 | synaptopodin 2 | NM_001128933 |
| **17013782** | 3,131 | 7,43E-03 | --- | --- | --- | NONHSAT115563 |
| **16912362** | 3,110 | 1,55E-03 | NM_002165 | ID1 | inhibitor of DNA binding 1, dominant negative helix-loop-helix protein | NM_002165 |
| **16753458** | 3,109 | 8,36E-03 | --- | --- | --- | ENST00000541870 |
| **17021062** | 3,094 | 1,12E-04 | NM_001122769 | LCA5 | Leber congenital amaurosis 5 | NM_001122769 |
| **16833136** | 3,090 | 1,44E-03 | --- | --- | --- | ENST00000584721 |
| **17112187** | 3,090 | 7,67E-03 | NR_030398 | MIR421 | microRNA 421 | NR_030398 |
| **16889249** | 3,075 | 4,41E-03 | --- | --- | --- | NONHSAT076307 |
| **17019749** | 3,074 | 3,39E-03 | NM_001098518 | ADGRF5 | adhesion G protein-coupled receptor F5 | NM_001098518 |
| **16675045** | 3,071 | 1,05E-04 | NM_031935 | HMCN1 | hemicentin 1 | NM_031935 |
| **17005573** | 3,054 | 2,70E-03 | NM_021063 | HIST1H2BD | histone cluster 1, H2bd | NM_021063 |
| **16707631** | 3,025 | 4,57E-03 | NM_001165979 | PLCE1 | phospholipase C, epsilon 1 | NM_001165979 |
| **16673229** | 3,021 | 2,44E-05 | --- | --- | --- | ENST00000558796 |
| **16978976** | 3,017 | 1,22E-02 | NM_000204 | CFI | complement factor I | NM_000204 |
| **17118392** | 3,015 | 7,89E-04 | AK130915 | LOC648570 | uncharacterized LOC648570 | AK130915 |
| **16853879** | 3,006 | 1,34E-05 | NM_022068 | PIEZO2 | piezo-type mechanosensitive ion channel component 2 | NM_022068 |
| **16868219** | 3,004 | 8,22E-04 | NM_001282352 | ADAMTS10 | ADAM metallopeptidase with thrombospondin type 1 motif 10 | NM_001282352 |
| **16677254** | 3,003 | 1,07E-03 | NR_004389 | SNORA16B | small nucleolar RNA, H/ACA box 16B | NR_004389 |
| **16996941** | 2,980 | 8,59E-03 | NR_027439 | LOC100272216 | uncharacterized LOC100272216 | NR_027439 |
| **16786650** | 2,967 | 1,02E-02 | --- | --- | --- | ENST00000364226 |
| **16747954** | 2,965 | 2,15E-03 | --- | --- | --- | NONHSAT026328 |
| **16945472** | 2,965 | 2,15E-03 | --- | --- | --- | NONHSAT026328 |
| **17074259** | 2,965 | 2,82E-02 | NM_001118887 | ANGPT2 | angiopoietin 2 | NM_001118887 |
| **16669630** | 2,961 | 5,27E-03 | --- | --- | --- | ENST00000614085 |
| **16669481** | 2,961 | 6,87E-04 | --- | --- | --- | ENST00000617273 |
| **17118142** | 2,934 | 5,37E-04 | XM_011543785 | LOC105379177 | uncharacterized LOC105379177 | XM_011543785 |
| **16673227** | 2,923 | 5,12E-04 | --- | --- | --- | ENST00000384354 |
| **16669501** | 2,921 | 6,34E-03 | XR_951295 | LOC105379521 | uncharacterized LOC105379521 | XR_951295 |
| **17112184** | 2,915 | 2,38E-03 | --- | --- | --- | ENST00000603037 |
| **16813590** | 2,912 | 1,40E-03 | AK128633 | LOC100130976 | uncharacterized LOC100130976 | AK128633 |
| **16977756** | 2,910 | 2,36E-04 | --- | --- | --- | NONHSAT097278 |
| **17101276** | 2,901 | 1,58E-03 | --- | --- | --- | NONHSAT136110 |
| **16884335** | 2,875 | 3,97E-03 | NM_001204106 | BCL2L11 | BCL2-like 11 (apoptosis facilitator) | NM_001204106 |
| **17010459** | 2,873 | 3,32E-03 | --- | --- | --- | ENST00000516689 |
| **16923763** | 2,869 | 2,62E-04 | --- | --- | --- | NONHSAT082954 |
| **17083319** | 2,868 | 3,57E-04 | NM_004972 | JAK2 | Janus kinase 2 | NM_004972 |
| **16712936** | 2,853 | 1,43E-04 | NR_030634 | MIR938 | microRNA 938 | NR_030634 |
| **16761502** | 2,851 | 3,15E-02 | NM_023922 | TAS2R14 | taste receptor, type 2, member 14 | NM_023922 |
| **16960911** | 2,840 | 7,44E-03 | NM_020169 | LXN | latexin | NM_020169 |
| **16835158** | 2,837 | 1,12E-04 | NM_000212 | ITGB3 | integrin beta 3 | NM_000212 |
| **16986913** | 2,834 | 3,48E-04 | NM_001126336 | VCAN | versican | NM_001126336 |
| **16959805** | 2,833 | 1,29E-03 | NM_001130992 | RBP1 | retinol binding protein 1, cellular | NM_001130992 |
| **16709358** | 2,830 | 1,25E-02 | --- | --- | --- | AK025137 |
| **17088098** | 2,820 | 1,25E-02 | --- | --- | --- | ENST00000384180 |
| **16965708** | 2,815 | 1,74E-02 | --- | --- | --- | ENST00000364129 |
| **16778043** | 2,810 | 4,36E-04 | --- | --- | --- | ENST00000456087 |
| **16756222** | 2,797 | 6,49E-03 | AB586692 | IGANRP | uncharacterized LOC100652933 | AB586692 |
| **16780592** | 2,786 | 3,66E-04 | NM_004951 | GPR183 | G protein-coupled receptor 183 | NM_004951 |
| **16802038** | 2,783 | 3,95E-04 | --- | --- | --- | ENST00000558443 |
| **16707047** | 2,773 | 5,83E-03 | XM_011539982 | PTEN | phosphatase and tensin homolog | XM_011539982 |
| **16778938** | 2,771 | 5,06E-03 | NM_000621 | HTR2A | 5-hydroxytryptamine (serotonin) receptor 2A, G protein-coupled | NM_000621 |
| **16755900** | 2,764 | 3,82E-02 | --- | --- | --- | NONHSAT030293 |
| **16911336** | 2,762 | 2,34E-03 | NR_004386 | RNU105B | RNA, U105B small nucleolar | NR_004386 |
| **16813565** | 2,759 | 9,77E-04 | NR_102743 | NR2F2-AS1 | NR2F2 antisense RNA 1 | NR_102743 |
| **16672298** | 2,758 | 2,20E-02 | --- | --- | --- | ENST00000419415 |
| **17055426** | 2,758 | 6,84E-03 | NM_001004320 | AGMO | alkylglycerol monooxygenase | NM_001004320 |
| **16888956** | 2,753 | 4,89E-02 | --- | --- | --- | ENST00000516321 |
| **16870782** | 2,749 | 8,80E-03 | NM_001159293 | ZNF737 | zinc finger protein 737 | NM_001159293 |
| **16770780** | 2,747 | 4,19E-02 | --- | --- | --- | ENST00000549373 |
| **16751655** | 2,742 | 3,99E-02 | NM_002178 | IGFBP6 | insulin like growth factor binding protein 6 | NM_002178 |
| **17118384** | 2,720 | 2,52E-03 | XM_011519130 | C9orf3 | chromosome 9 open reading frame 3 | XM_011519130 |
| **16870401** | 2,719 | 1,45E-02 | NM_001170938 | ISYNA1 | inositol-3-phosphate synthase 1 | NM_001170938 |
| **16942917** | 2,690 | 1,04E-03 | --- | --- | --- | ENST00000408132 |
| **16971272** | 2,685 | 2,78E-03 | NM_001166055 | EDNRA | endothelin receptor type A | NM_001166055 |
| **16999475** | 2,679 | 4,21E-04 | NM_001999 | FBN2 | fibrillin 2 | NM_001999 |
| **16881350** | 2,675 | 2,26E-02 | BC044944 | ZNF638-IT1 | ZNF638 intronic transcript 1 | BC044944 |
| **16829618** | 2,658 | 1,15E-03 | NM_001098202 | HIC1 | hypermethylated in cancer 1 | NM_001098202 |
| **16664608** | 2,646 | 8,72E-04 | --- | --- | --- | NONHSAT003192 |
| **17012447** | 2,644 | 1,11E-02 | NM_000426 | LAMA2 | laminin, alpha 2 | NM_000426 |
| **16667516** | 2,633 | 3,04E-02 | NM_001166252 | PLPPR4 | phospholipid phosphatase related 4 | NM_001166252 |
| **16894283** | 2,609 | 6,56E-03 | NM_138799 | MBOAT2 | membrane bound O-acyltransferase domain containing 2 | NM_138799 |
| **16831550** | 2,600 | 8,84E-03 | NR_026810 | FAM106CP | family with sequence similarity 106, member C, pseudogene | NR_026810 |
| **17080648** | 2,599 | 9,04E-03 | NM_005328 | HAS2 | hyaluronan synthase 2 | NM_005328 |
| **16776045** | 2,599 | 2,59E-02 | --- | --- | --- | NONHSAT034911 |
| **17009760** | 2,597 | 3,01E-04 | NM_001010872 | FAM83B | family with sequence similarity 83, member B | NM_001010872 |
| **16878541** | 2,594 | 1,64E-02 | ENST00000462832 | SPDYA | speedy/RINGO cell cycle regulator family member A | ENST00000462832 |
| **16889954** | 2,594 | 1,40E-03 | NR_002589 | SNORD51 | small nucleolar RNA, C/D box 51 | NR_002589 |
| **17102129** | 2,592 | 5,42E-03 | NM_002970 | SAT1 | spermidine/spermine N1-acetyltransferase 1 | NM_002970 |
| **16884372** | 2,592 | 8,50E-03 | NM_006343 | MERTK | MER proto-oncogene, tyrosine kinase | NM_006343 |
| **16832835** | 2,590 | 3,32E-02 | BC047718 | SUZ12P1 | SUZ12 polycomb repressive complex 2 subunit pseudogene 1 | BC047718 |
| **17088116** | 2,583 | 4,60E-02 | NR_039814 | MIR4668 | microRNA 4668 | NR_039814 |
| **16858710** | 2,579 | 6,22E-05 | NM_002229 | JUNB | jun B proto-oncogene | NM_002229 |
| **16938407** | 2,579 | 1,25E-02 | NM_000965 | RARB | retinoic acid receptor, beta | NM_000965 |
| **16880168** | 2,569 | 2,72E-03 | NR_002229 | RPL23AP32 | ribosomal protein L23a pseudogene 32 | NR_002229 |
| **16884364** | 2,563 | 1,33E-02 | --- | --- | --- | AK092410 |
| **16950171** | 2,541 | 1,02E-02 | --- | --- | --- | ENST00000364752 |
| **16798080** | 2,534 | 2,16E-03 | --- | --- | --- | ENST00000551631 |
| **16773032** | 2,529 | 3,27E-02 | --- | --- | --- | ENST00000410794 |
| **17008209** | 2,520 | 1,45E-02 | --- | --- | --- | ENST00000363727 |
| **16882536** | 2,520 | 9,20E-03 | --- | --- | --- | --- |
| **17070110** | 2,510 | 3,36E-02 | NM_015886 | PI15 | peptidase inhibitor 15 | NM_015886 |
| **16986417** | 2,504 | 6,49E-03 | NM_005242 | F2RL1 | coagulation factor II (thrombin) receptor-like 1 | NM_005242 |
| **16689869** | 2,501 | 8,12E-04 | NM_001178096 | F3 | coagulation factor III (thromboplastin, tissue factor) | NM_001178096 |
| **16997799** | 2,494 | 8,28E-03 | --- | --- | --- | ENST00000513899 |
| **16874265** | 2,493 | 8,72E-04 | NR_036209 | MIR4324 | microRNA 4324 | NR_036209 |
| **17117463** | 2,489 | 2,51E-02 | NM_001282736 | PLXDC2 | plexin domain containing 2 | NM_001282736 |
| **16703036** | 2,487 | 1,04E-02 | NM_001282736 | PLXDC2 | plexin domain containing 2 | NM_001282736 |
| **16852568** | 2,482 | 1,64E-02 | --- | --- | --- | ENST00000365289 |
| **16828270** | 2,481 | 1,04E-03 | --- | --- | --- | NONHSAT143685 |
| **16948572** | 2,478 | 2,69E-02 | NM_017644 | KLHL24 | kelch-like family member 24 | NM_017644 |
| **16714725** | 2,458 | 7,67E-05 | NM_178505 | TMEM26 | transmembrane protein 26 | NM_178505 |
| **17117655** | 2,455 | 6,48E-04 | AK130544 | LOC100507079 | uncharacterized LOC100507079 | AK130544 |
| **16829683** | 2,451 | 3,92E-03 | --- | --- | --- | NONHSAT144976 |
| **16972098** | 2,449 | 3,81E-02 | --- | --- | --- | ENST00000411105 |
| **16705437** | 2,447 | 2,38E-02 | NR_031655 | MIR1254-1 | microRNA 1254-1 | NR_031655 |
| **16852966** | 2,446 | 3,53E-03 | NM_001093729 | CCDC102B | coiled-coil domain containing 102B | NM_001093729 |
| **16785083** | 2,440 | 1,56E-02 | --- | --- | --- | --- |
| **16825055** | 2,439 | 6,06E-04 | --- | --- | --- | NONHSAT141107 |
| **17024667** | 2,437 | 7,40E-03 | --- | --- | --- | --- |
| **16910070** | 2,435 | 7,26E-03 | NR_039643 | MIR4441 | microRNA 4441 | NR_039643 |
| **16784787** | 2,434 | 2,04E-03 | NM_001270520 | DAAM1 | dishevelled associated activator of morphogenesis 1 | NM_001270520 |
| **16851822** | 2,431 | 4,27E-02 | --- | --- | --- | NONHSAT058854 |
| **16702501** | 2,429 | 2,33E-02 | --- | --- | --- | NONHSAT011447 |
| **16988913** | 2,406 | 1,52E-02 | --- | --- | --- | ENST00000408635 |
| **16943467** | 2,399 | 3,10E-03 | NM_001005474 | NFKBIZ | nuclear factor of kappa light polypeptide gene enhancer in B-cells inhibitor, zeta | NM_001005474 |
| **17117880** | 2,392 | 1,33E-02 | --- | --- | --- | AK130262 |
| **16674973** | 2,381 | 1,81E-03 | NM_030806 | C1orf21 | chromosome 1 open reading frame 21 | NM_030806 |
| **16996983** | 2,378 | 3,82E-04 | NM_004536 | NAIP | NLR family, apoptosis inhibitory protein | NM_004536 |
| **16840766** | 2,357 | 9,59E-03 | AK128353 | LOC100128281 | uncharacterized LOC100128281 | AK128353 |
| **16882576** | 2,355 | 2,62E-04 | NR_039634 | MIR4435-1 | microRNA 4435-1 | NR_039634 |
| **16901825** | 2,355 | 2,62E-04 | NR_039634 | MIR4435-1 | microRNA 4435-1 | NR_039634 |
| **16947041** | 2,352 | 2,68E-02 | XR_924585 | LOC105374160 | uncharacterized LOC105374160 | XR_924585 |
| **17052417** | 2,345 | 4,01E-02 | NM_016944 | TAS2R4 | taste receptor, type 2, member 4 | NM_016944 |
| **16843906** | 2,341 | 7,23E-03 | XM_006722254 | TBC1D3E | TBC1 domain family, member 3E | XM_006722254 |
| **16742738** | 2,340 | 3,30E-04 | ENST00000529798 | TENM4 | teneurin transmembrane protein 4 | ENST00000529798 |
| **16870774** | 2,339 | 2,54E-02 | NR_036053 | MIR1270 | microRNA 1270 | NR_036053 |
| **16870780** | 2,339 | 2,54E-02 | NR_036053 | MIR1270 | microRNA 1270 | NR_036053 |
| **16756158** | 2,334 | 1,81E-03 | --- | --- | --- | ENST00000362545 |
| **17096728** | 2,325 | 1,12E-04 | NM_005502 | ABCA1 | ATP binding cassette subfamily A member 1 | NM_005502 |
| **16877760** | 2,322 | 2,04E-02 | --- | --- | --- | ENST00000384005 |
| **17050350** | 2,321 | 4,69E-02 | NM_001099658 | LRRN3 | leucine rich repeat neuronal 3 | NM_001099658 |
| **16798071** | 2,315 | 7,76E-03 | --- | --- | --- | NONHSAT040988 |
| **16919247** | 2,304 | 1,42E-04 | XR_920082 | LOC101927098 | uncharacterized LOC101927098 | XR_920082 |
| **16778005** | 2,294 | 2,58E-05 | NM_001243466 | STARD13 | StAR-related lipid transfer domain containing 13 | NM_001243466 |
| **17019728** | 2,292 | 1,27E-02 | NM_001168357 | PLA2G7 | phospholipase A2, group VII (platelet-activating factor acetylhydrolase, plasma) | NM_001168357 |
| **16967863** | 2,289 | 2,10E-03 | ENST00000511560 | AREG | amphiregulin | ENST00000511560 |
| **17005276** | 2,289 | 4,96E-03 | NM_003107 | SOX4 | SRY box 4 | NM_003107 |
| **16750254** | 2,288 | 4,96E-03 | --- | --- | --- | ENST00000363016 |
| **17014157** | 2,285 | 1,14E-03 | NR_046796 | SYNJ2-IT1 | SYNJ2 intronic transcript 1 | NR_046796 |
| **16846081** | 2,285 | 7,22E-03 | --- | --- | --- | ENST00000523101 |
| **16774384** | 2,278 | 3,15E-03 | NM_003701 | TNFSF11 | tumor necrosis factor (ligand) superfamily, member 11 | NM_003701 |
| **16802601** | 2,271 | 1,09E-02 | --- | --- | --- | ENST00000558429 |
| **16779701** | 2,270 | 3,12E-04 | NM_004392 | DACH1 | dachshund family transcription factor 1 | NM_004392 |
| **17047578** | 2,269 | 2,14E-02 | NR_029411 | LOC100133091 | uncharacterized LOC100133091 | NR_029411 |
| **16748933** | 2,269 | 1,89E-02 | --- | --- | --- | ENST00000501211 |
| **16742384** | 2,267 | 3,81E-03 | NM_001128922 | LRRC32 | leucine rich repeat containing 32 | NM_001128922 |
| **17058812** | 2,266 | 1,89E-02 | --- | --- | --- | --- |
| **17062457** | 2,265 | 1,25E-02 | --- | --- | --- | NONHSAT123061 |
| **16821777** | 2,263 | 5,22E-03 | --- | --- | --- | NONHSAT144362 |
| **16704977** | 2,257 | 1,18E-02 | NM_001098512 | PRKG1 | protein kinase, cGMP-dependent, type I | NM_001098512 |
| **16967853** | 2,252 | 2,55E-03 | NM_001657 | AREG | amphiregulin | NM_001657 |
| **16673191** | 2,252 | 1,80E-03 | NM_001204961 | PBX1 | pre-B-cell leukemia homeobox 1 | NM_001204961 |
| **16802232** | 2,250 | 6,22E-05 | NM_005585 | SMAD6 | SMAD family member 6 | NM_005585 |
| **16826389** | 2,247 | 1,93E-02 | NM_001271620 | ZNF423 | zinc finger protein 423 | NM_001271620 |
| **17047367** | 2,246 | 1,16E-02 | --- | --- | --- | NONHSAT121380 |
| **17058392** | 2,246 | 1,16E-02 | --- | --- | --- | NONHSAT121380 |
| **16879500** | 2,245 | 1,16E-03 | NM_172069 | PLEKHH2 | pleckstrin homology domain containing, family H (with MyTH4 domain) member 2 | NM_172069 |
| **16781352** | 2,241 | 1,70E-03 | --- | --- | --- | ENST00000459523 |
| **16901827** | 2,237 | 2,16E-02 | --- | --- | --- | ENST00000432268 |
| **17102297** | 2,234 | 4,05E-02 | NM_001136533 | DCAF8L2 | DDB1 and CUL4 associated factor 8-like 2 | NM_001136533 |
| **16999472** | 2,232 | 1,63E-02 | ENST00000512185 | LINC01184 | long intergenic non-protein coding RNA 1184 | ENST00000512185 |
| **16995336** | 2,228 | 1,98E-02 | --- | --- | --- | NONHSAT101049 |
| **16900187** | 2,227 | 8,92E-03 | --- | --- | --- | --- |
| **17045182** | 2,222 | 3,96E-03 | NM_030636 | EEPD1 | endonuclease/exonuclease/phosphatase family domain containing 1 | NM_030636 |
| **16998059** | 2,221 | 7,02E-03 | NM_020801 | ARRDC3 | arrestin domain containing 3 | NM_020801 |
| **16848219** | 2,213 | 2,33E-02 | NM_080284 | ABCA6 | ATP binding cassette subfamily A member 6 | NM_080284 |
| **16906285** | 2,212 | 3,46E-02 | NM_001271751 | CALCRL | calcitonin receptor like receptor | NM_001271751 |
| **17058121** | 2,206 | 3,24E-02 | NM_001130022 | ZNF680 | zinc finger protein 680 | NM_001130022 |
| **16932988** | 2,205 | 1,33E-02 | NR_003950 | ZDHHC8P1 | zinc finger, DHHC-type containing 8 pseudogene 1 | NR_003950 |
| **16960807** | 2,201 | 5,45E-03 | NM_001308185 | CCNL1 | cyclin L1 | NM_001308185 |
| **16852371** | 2,200 | 2,10E-02 | ENST00000591126 | SMAD4 | SMAD family member 4 | ENST00000591126 |
| **16994303** | 2,182 | 3,30E-04 | --- | --- | --- | ENST00000515377 |
| **16905495** | 2,181 | 2,32E-02 | --- | --- | --- | AK128852 |
| **17043177** | 2,176 | 7,31E-03 | X58060 | SNORD13P2 | small nucleolar RNA, C/D box 13 pseudogene 2 | X58060 |
| **17087633** | 2,176 | 4,96E-03 | NM_001278231 | ZNF189 | zinc finger protein 189 | NM_001278231 |
| **16748888** | 2,175 | 1,01E-03 | NM_001143821 | PLEKHA5 | pleckstrin homology domain containing, family A member 5 | NM_001143821 |
| **16678518** | 2,171 | 3,30E-03 | NM_021205 | RHOU | ras homolog family member U | NM_021205 |
| **16856172** | 2,166 | 8,36E-03 | --- | --- | --- | ENST00000516730 |
| **17088991** | 2,158 | 8,06E-04 | NR_038975 | MIR181A2HG | MIR181A2 host gene | NR_038975 |
| **16824865** | 2,150 | 2,53E-02 | OTTHUMT00000403182 | NPIPB5 | nuclear pore complex interacting protein family, member B5 | OTTHUMT00000403182 |
| **16921464** | 2,149 | 1,25E-02 | XR_424059 | LOC101930100 | uncharacterized LOC101930100 | XR_424059 |
| **16940691** | 2,146 | 1,94E-02 | --- | --- | --- | NONHSAT089687 |
| **16744770** | 2,146 | 2,65E-02 | AK058022 | SIK3-IT1 | SIK3 intronic transcript 1 | AK058022 |
| **17004198** | 2,145 | 8,73E-03 | NM_001452 | FOXF2 | forkhead box F2 | NM_001452 |
| **17085376** | 2,137 | 2,73E-02 | XR_929680 | LOC105376066 | uncharacterized LOC105376066 | XR_929680 |
| **16798073** | 2,135 | 1,88E-02 | AK096584 | PWAR6 | Prader Willi/Angelman region RNA 6 | AK096584 |
| **16816897** | 2,134 | 2,68E-02 | OTTHUMT00000402426 | NPIPB4 | nuclear pore complex interacting protein family, member B4 | OTTHUMT00000402426 |
| **16824056** | 2,130 | 1,22E-03 | --- | --- | --- | AB586691 |
| **17064600** | 2,126 | 3,41E-03 | --- | --- | --- | ENST00000424630 |
| **16684785** | 2,125 | 2,68E-02 | NM_001171940 | FNDC5 | fibronectin type III domain containing 5 | NM_001171940 |
| **16744353** | 2,123 | 1,02E-02 | --- | --- | --- | ENST00000531305 |
| **16872551** | 2,115 | 1,00E-03 | NM_000660 | TGFB1 | transforming growth factor beta 1 | NM_000660 |
| **16798696** | 2,114 | 4,27E-02 | --- | --- | --- | ENST00000384701 |
| **17003640** | 2,113 | 2,10E-03 | NM_014244 | ADAMTS2 | ADAM metallopeptidase with thrombospondin type 1 motif 2 | NM_014244 |
| **16928428** | 2,110 | 9,13E-04 | NM_005160 | ADRBK2 | adrenergic, beta, receptor kinase 2 | NM_005160 |
| **16667702** | 2,109 | 7,27E-03 | NM_001078 | VCAM1 | vascular cell adhesion molecule 1 | NM_001078 |
| **16701269** | 2,108 | 2,07E-02 | --- | --- | --- | ENST00000417120 |
| **16959007** | 2,105 | 1,25E-03 | NM_015103 | PLXND1 | plexin D1 | NM_015103 |
| **16707030** | 2,105 | 1,23E-03 | NM_000314 | PTEN | phosphatase and tensin homolog | NM_000314 |
| **17104363** | 2,104 | 7,31E-03 | NM_004429 | EFNB1 | ephrin-B1 | NM_004429 |
| **16787902** | 2,096 | 1,06E-02 | NM_001085 | SERPINA3 | serpin peptidase inhibitor, clade A (alpha-1 antiproteinase, antitrypsin), member 3 | NM_001085 |
| **16990949** | 2,095 | 3,51E-03 | NR_029684 | MIR143 | microRNA 143 | NR_029684 |
| **16675840** | 2,093 | 4,07E-02 | --- | --- | --- | ENST00000364072 |
| **16967319** | 2,091 | 7,02E-03 | --- | --- | --- | ENST00000365504 |
| **17008651** | 2,088 | 2,20E-02 | --- | --- | --- | ENST00000384121 |
| **17084959** | 2,088 | 4,23E-02 | --- | --- | --- | NONHSAT131047 |
| **16673652** | 2,088 | 1,26E-03 | AK130711 | LOC100127910 | uncharacterized LOC100127910 | AK130711 |
| **16923766** | 2,086 | 1,59E-02 | NM_030582 | COL18A1 | collagen, type XVIII, alpha 1 | NM_030582 |
| **16942458** | 2,085 | 4,10E-02 | NR_038264 | ADAMTS9-AS2 | ADAMTS9 antisense RNA 2 | NR_038264 |
| **16985823** | 2,084 | 1,42E-02 | NM_000344 | SMN1 | survival of motor neuron 1, telomeric | NM_000344 |
| **16949499** | 2,083 | 2,37E-03 | NR_024413 | FLJ42393 | uncharacterized LOC401105 | NR_024413 |
| **17098567** | 2,083 | 1,65E-02 | NM_001142531 | SH2D3C | SH2 domain containing 3C | NM_001142531 |
| **16852369** | 2,078 | 6,89E-03 | ENST00000585448 | SMAD4 | SMAD family member 4 | ENST00000585448 |
| **16700806** | 2,073 | 6,27E-03 | NM_000081 | LYST | lysosomal trafficking regulator | NM_000081 |
| **16775811** | 2,068 | 1,73E-02 | NM_005708 | GPC6 | glypican 6 | NM_005708 |
| **17077051** | 2,065 | 8,31E-03 | NM_001286782 | PCMTD1 | protein-L-isoaspartate (D-aspartate) O-methyltransferase domain containing 1 | NM_001286782 |
| **16819736** | 2,064 | 2,70E-03 | --- | --- | --- | NONHSAT142868 |
| **16666929** | 2,062 | 2,35E-02 | --- | --- | --- | ENST00000384505 |
| **16888270** | 2,060 | 1,33E-02 | NM_000885 | ITGA4 | integrin alpha 4 | NM_000885 |
| **16806511** | 2,059 | 6,26E-03 | --- | --- | --- | ENST00000560740 |
| **17024079** | 2,050 | 4,92E-03 | NM_005923 | MAP3K5 | mitogen-activated protein kinase kinase kinase 5 | NM_005923 |
| **16692632** | 2,047 | 4,28E-02 | NM_003528 | HIST2H2BE | histone cluster 2, H2be | NM_003528 |
| **16900724** | 2,044 | 1,89E-02 | uc002sxv.4 | LOC100506123 | uncharacterized LOC100506123 | uc002sxv.4 |
| **16917183** | 2,042 | 1,22E-02 | NM_000214 | JAG1 | jagged 1 | NM_000214 |
| **16687875** | 2,035 | 1,52E-02 | NM_002228 | JUN | jun proto-oncogene | NM_002228 |
| **16985759** | 2,034 | 1,69E-02 | XR_427728 | LOC102724275 | uncharacterized LOC102724275 | XR_427728 |
| **16863423** | 2,031 | 1,45E-02 | NM_022462 | HIF3A | hypoxia inducible factor 3, alpha subunit | NM_022462 |
| **16956053** | 2,026 | 1,36E-02 | NR_036087 | MIR3136 | microRNA 3136 | NR_036087 |
| **17010539** | 2,025 | 3,53E-03 | XM_011536282 | LOC105377866 | uncharacterized LOC105377866 | XM_011536282 |
| **16882226** | 2,022 | 4,71E-02 | --- | --- | --- | NONHSAT071992 |
| **16753399** | 2,019 | 8,52E-03 | --- | --- | --- | ENST00000550290 |
| **16778274** | 2,017 | 3,02E-02 | NM_001135955 | TRPC4 | transient receptor potential cation channel, subfamily C, member 4 | NM_001135955 |
| **16918102** | 2,014 | 5,08E-03 | NM_025176 | NINL | ninein-like | NM_025176 |
| **16718592** | 2,014 | 2,80E-02 | NM_001001936 | AFAP1L2 | actin filament associated protein 1-like 2 | NM_001001936 |
| **17076634** | 2,013 | 5,77E-04 | NM_000037 | ANK1 | ankyrin 1, erythrocytic | NM_000037 |
| **16869596** | 2,012 | 4,96E-03 | NM_001008701 | ADGRL1 | adhesion G protein-coupled receptor L1 | NM_001008701 |
| **16821541** | 2,010 | 2,64E-02 | NM_031476 | CRISPLD2 | cysteine-rich secretory protein LCCL domain containing 2 | NM_031476 |
| **16691619** | 2,009 | 8,22E-04 | NR_036540 | LINC00622 | long intergenic non-protein coding RNA 622 | NR_036540 |
| **16997046** | 2,006 | 1,10E-02 | NR_033968 | GUSBP9 | glucuronidase, beta pseudogene 9 | NR_033968 |
| **16728883** | 2,006 | 3,95E-03 | --- | --- | --- | ENST00000538624 |
| **16727700** | 2,002 | 1,06E-02 | --- | --- | --- | NONHSAT022381 |
| **16731654** | 0,500 | 1,69E-03 | NM_001001522 | TAGLN | transgelin | NM_001001522 |
| **16983765** | 0,499 | 3,15E-03 | NM_001204375 | NPR3 | natriuretic peptide receptor C/guanylate cyclase C (atrionatriuretic peptide receptor C) | NM_001204375 |
| **16729290** | 0,499 | 1,70E-03 | NM_001258210 | TSKU | tsukushi, small leucine rich proteoglycan | NM_001258210 |
| **17096471** | 0,499 | 8,28E-03 | NM_001267571 | TBC1D2 | TBC1 domain family, member 2 | NM_001267571 |
| **16727427** | 0,499 | 2,35E-03 | NM_005700 | DPP3 | dipeptidyl-peptidase 3 | NM_005700 |
| **16688506** | 0,499 | 2,20E-02 | ENST00000357731 | NEGR1 | neuronal growth regulator 1 | ENST00000357731 |
| **17083261** | 0,498 | 4,96E-03 | NM_004170 | SLC1A1 | solute carrier family 1, member 1 | NM_004170 |
| **16947357** | 0,497 | 2,62E-01 | NM_002852 | PTX3 | pentraxin 3, long | NM_002852 |
| **16875754** | 0,496 | 2,02E-03 | NM_000641 | IL11 | interleukin 11 | NM_000641 |
| **16682348** | 0,496 | 1,14E-03 | NM_022089 | ATP13A2 | ATPase type 13A2 | NM_022089 |
| **17116681** | 0,495 | 3,24E-02 | NM_005840 | SPRY3 | sprouty homolog 3 (Drosophila) | NM_005840 |
| **16662209** | 0,493 | 1,81E-03 | NM_052998 | ADC | arginine decarboxylase | NM_052998 |
| **16885859** | 0,493 | 3,61E-04 | --- | --- | --- | ENST00000432414 |
| **16778713** | 0,493 | 5,06E-03 | --- | --- | --- | ENST00000517242 |
| **16984287** | 0,492 | 8,12E-04 | ENST00000302472 | PTGER4 | prostaglandin E receptor 4 (subtype EP4) | ENST00000302472 |
| **16941691** | 0,491 | 9,73E-03 | NR_003057 | SNORD69 | small nucleolar RNA, C/D box 69 | NR_003057 |
| **16855545** | 0,490 | 1,20E-02 | NM_052947 | ALPK2 | alpha-kinase 2 | NM_052947 |
| **16819233** | 0,490 | 1,75E-01 | NM_005946 | MT1A | metallothionein 1A | NM_005946 |
| **16902015** | 0,489 | 1,79E-03 | ENST00000429538 | PAX8 | paired box 8 | ENST00000429538 |
| **17000520** | 0,488 | 9,29E-02 | NR_002913 | SNORD63 | small nucleolar RNA, C/D box 63 | NR_002913 |
| **16984730** | 0,488 | 1,90E-04 | NM_006350 | FST | follistatin | NM_006350 |
| **16881353** | 0,487 | 2,01E-03 | NM_001130987 | DYSF | dysferlin, limb girdle muscular dystrophy 2B (autosomal recessive) | NM_001130987 |
| **17098506** | 0,487 | 2,87E-03 | NM_022833 | FAM129B | family with sequence similarity 129, member B | NM_022833 |
| **17012709** | 0,487 | 6,98E-04 | NR_002435 | SNORD100 | small nucleolar RNA, C/D box 100 | NR_002435 |
| **16986138** | 0,487 | 2,13E-04 | NM_001080479 | ARHGEF28 | Rho guanine nucleotide exchange factor (GEF) 28 | NM_001080479 |
| **16693474** | 0,486 | 1,08E-02 | NM_080388 | S100A16 | S100 calcium binding protein A16 | NM_080388 |
| **16842850** | 0,486 | 6,35E-02 | NM_144683 | DHRS13 | dehydrogenase/reductase (SDR family) member 13 | NM_144683 |
| **16696531** | 0,485 | 6,08E-03 | ENST00000385582 | SNORD78 | small nucleolar RNA, C/D box 78 | ENST00000385582 |
| **16918722** | 0,483 | 3,01E-02 | NM_000557 | GDF5 | growth differentiation factor 5 | NM_000557 |
| **17005138** | 0,483 | 3,80E-03 | ENST00000229922 | CAP2 | CAP, adenylate cyclase-associated protein, 2 (yeast) | ENST00000229922 |
| **16964098** | 0,482 | 2,16E-03 | NR_003004 | SCARNA22 | small Cajal body-specific RNA 22 | NR_003004 |
| **16716478** | 0,482 | 1,05E-02 | NM_014391 | ANKRD1 | ankyrin repeat domain 1 (cardiac muscle) | NM_014391 |
| **16845158** | 0,481 | 8,26E-04 | NM_012232 | PTRF | polymerase I and transcript release factor | NM_012232 |
| **16885189** | 0,481 | 2,14E-02 | NR_023343 | RNU4ATAC | RNA, U4atac small nuclear (U12-dependent splicing) | NR_023343 |
| **17101368** | 0,481 | 3,81E-03 | NM_001649 | SHROOM2 | shroom family member 2 | NM_001649 |
| **17112918** | 0,481 | 3,21E-02 | NM_018476 | BEX1 | brain expressed, X-linked 1 | NM_018476 |
| **16900136** | 0,479 | 1,25E-02 | --- | --- | --- | M85256 |
| **16896561** | 0,477 | 1,25E-02 | NM_000104 | CYP1B1 | cytochrome P450, family 1, subfamily B, polypeptide 1 | NM_000104 |
| **17088063** | 0,476 | 4,10E-03 | --- | --- | --- | GENSCAN00000039084 |
| **16658023** | 0,476 | 5,72E-03 | ENST00000378486 | PLCH2 | phospholipase C, eta 2 | ENST00000378486 |
| **16952769** | 0,474 | 3,91E-03 | NM_022842 | CDCP1 | CUB domain containing protein 1 | NM_022842 |
| **16664005** | 0,472 | 2,29E-03 | ENST00000372201 | PLK3 | polo-like kinase 3 | ENST00000372201 |
| **16943944** | 0,469 | 1,34E-01 | NM_001004196 | CD200 | CD200 molecule | NM_001004196 |
| **17080486** | 0,469 | 3,62E-03 | NM_002546 | TNFRSF11B | tumor necrosis factor receptor superfamily, member 11b | NM_002546 |
| **17094598** | 0,468 | 5,74E-02 | --- | --- | --- | ENST00000377421 |
| **16844872** | 0,468 | 2,09E-03 | NM_002230 | JUP | junction plakoglobin | NM_002230 |
| **16808563** | 0,467 | 1,00E-02 | --- | --- | --- | ENST00000560967 |
| **16725356** | 0,465 | 4,71E-04 | NM_024098 | CCDC86 | coiled-coil domain containing 86 | NM_024098 |
| **16868818** | 0,464 | 1,11E-02 | NR_029586 | MIR199A1 | microRNA 199a-1 | NR_029586 |
| **16785379** | 0,464 | 7,62E-03 | NM_021979 | HSPA2 | heat shock 70kDa protein 2 | NM_021979 |
| **16748529** | 0,464 | 4,25E-04 | ENST00000014914 | GPRC5A | G protein-coupled receptor, family C, group 5, member A | ENST00000014914 |
| **16715699** | 0,463 | 1,82E-03 | NM_001001791 | C10orf55 | chromosome 10 open reading frame 55 | NM_001001791 |
| **16849238** | 0,463 | 1,08E-03 | NM_024599 | RHBDF2 | rhomboid 5 homolog 2 (Drosophila) | NM_024599 |
| **16833629** | 0,462 | 2,18E-03 | NM_001199417 | ARHGAP23 | Rho GTPase activating protein 23 | NM_001199417 |
| **16976029** | 0,462 | 2,50E-02 | NM_002253 | KDR | kinase insert domain receptor (a type III receptor tyrosine kinase) | NM_002253 |
| **16776160** | 0,462 | 3,25E-03 | NM_001271754 | ITGBL1 | integrin, beta-like 1 (with EGF-like repeat domains) | NM_001271754 |
| **16675731** | 0,462 | 8,46E-03 | AY245430 | IGFN1 | immunoglobulin-like and fibronectin type III domain containing 1 | AY245430 |
| **16835589** | 0,457 | 1,05E-02 | --- | --- | --- | ENST00000506504 |
| **16967771** | 0,456 | 8,12E-04 | NM_000584 | IL8 | interleukin 8 | NM_000584 |
| **16986943** | 0,455 | 1,64E-02 | --- | --- | --- | ENST00000502253 |
| **17046524** | 0,454 | 3,36E-02 | --- | --- | --- | ENST00000384614 |
| **16855180** | 0,454 | 7,56E-03 | NR_003701 | SNORD58C | small nucleolar RNA, C/D box 58C | NR_003701 |
| **16740630** | 0,450 | 1,27E-02 | NM_005438 | FOSL1 | FOS-like antigen 1 | NM_005438 |
| **16807763** | 0,449 | 6,68E-03 | ENST00000220325 | EHD4 | EH-domain containing 4 | ENST00000220325 |
| **17096457** | 0,448 | 8,69E-03 | NM_003389 | CORO2A | coronin, actin binding protein, 2A | NM_003389 |
| **17096493** | 0,447 | 5,11E-03 | NM_005458 | GABBR2 | gamma-aminobutyric acid (GABA) B receptor, 2 | NM_005458 |
| **16819217** | 0,446 | 1,33E-01 | ENST00000306061 | MT1E | metallothionein 1E | ENST00000306061 |
| **16762470** | 0,445 | 2,21E-03 | NM_030762 | BHLHE41 | basic helix-loop-helix family, member e41 | NM_030762 |
| **17016221** | 0,442 | 2,92E-03 | NM_014722 | FAM65B | family with sequence similarity 65, member B | NM_014722 |
| **16798198** | 0,441 | 7,60E-04 | NR_003332 | SNORD116-17 | small nucleolar RNA, C/D box 116-17 | NR_003332 |
| **16798202** | 0,441 | 7,60E-04 | NR_003332 | SNORD116-17 | small nucleolar RNA, C/D box 116-17 | NR_003332 |
| **16709856** | 0,439 | 7,64E-05 | NM_004281 | BAG3 | BCL2-associated athanogene 3 | NM_004281 |
| **16788776** | 0,439 | 5,12E-04 | NR_030160 | MIR485 | microRNA 485 | NR_030160 |
| **16788764** | 0,439 | 7,31E-03 | NR_030257 | MIR544A | microRNA 544a | NR_030257 |
| **16718670** | 0,439 | 2,52E-03 | NM_005264 | GFRA1 | GDNF family receptor alpha 1 | NM_005264 |
| **16677451** | 0,437 | 4,28E-03 | NM_001017424 | KCNK2 | potassium channel, subfamily K, member 2 | NM_001017424 |
| **17093861** | 0,437 | 2,52E-03 | NM_001044264 | MSMP | microseminoprotein, prostate associated | NM_001044264 |
| **16685868** | 0,436 | 1,73E-02 | NM_133467 | CITED4 | Cbp/p300-interacting transactivator, with Glu/Asp-rich carboxy-terminal domain, 4 | NM_133467 |
| **16704154** | 0,434 | 8,73E-03 | ENST00000355710 | RET | ret proto-oncogene | ENST00000355710 |
| **16811638** | 0,433 | 7,15E-03 | NM_003612 | SEMA7A | semaphorin 7A, GPI membrane anchor (John Milton Hagen blood group) | NM_003612 |
| **17012761** | 0,431 | 2,43E-03 | ENST00000367882 | TCF21 | transcription factor 21 | ENST00000367882 |
| **17002667** | 0,429 | 1,90E-02 | NM_001129891 | FAM196B | family with sequence similarity 196, member B | NM_001129891 |
| **16869291** | 0,428 | 1,07E-02 | NR_002751 | SNORD41 | small nucleolar RNA, C/D box 41 | NR_002751 |
| **16670183** | 0,427 | 1,33E-01 | --- | --- | --- | ENST00000363009 |
| **17002898** | 0,427 | 2,36E-04 | NM_003714 | STC2 | stanniocalcin 2 | NM_003714 |
| **16788796** | 0,427 | 2,04E-03 | NR_030392 | MIR656 | microRNA 656 | NR_030392 |
| **16897493** | 0,427 | 1,96E-02 | --- | --- | --- | n365802 |
| **17113488** | 0,425 | 2,15E-03 | --- | --- | --- | HQ292112 |
| **16777571** | 0,424 | 3,23E-03 | NM_001007538 | SHISA2 | shisa homolog 2 (Xenopus laevis) | NM_001007538 |
| **16788780** | 0,423 | 1,42E-02 | NR_029704 | MIR154 | microRNA 154 | NR_029704 |
| **16917849** | 0,423 | 6,81E-03 | ENST00000377103 | THBD | thrombomodulin | ENST00000377103 |
| **16852871** | 0,423 | 3,67E-02 | NM_001143818 | SERPINB2 | serpin peptidase inhibitor, clade B (ovalbumin), member 2 | NM_001143818 |
| **17110367** | 0,422 | 9,59E-03 | NR_029636 | MIR222 | microRNA 222 | NR_029636 |
| **16706180** | 0,420 | 6,22E-05 | ENST00000446342 | PLAU | plasminogen activator, urokinase | ENST00000446342 |
| **16788751** | 0,418 | 3,04E-03 | NR_031571 | MIR1185-2 | microRNA 1185-2 | NR_031571 |
| **17087109** | 0,417 | 3,01E-03 | NR_029665 | MIR27B | microRNA 27b | NR_029665 |
| **16788023** | 0,417 | 3,74E-02 | NM_000623 | BDKRB2 | bradykinin receptor B2 | NM_000623 |
| **17016110** | 0,414 | 3,25E-03 | NM_016356 | DCDC2 | doublecortin domain containing 2 | NM_016356 |
| **16964676** | 0,414 | 1,62E-05 | NM_006005 | WFS1 | Wolfram syndrome 1 (wolframin) | NM_006005 |
| **16822892** | 0,413 | 6,40E-03 | NR_002326 | SNORA64 | small nucleolar RNA, H/ACA box 64 | NR_002326 |
| **16697660** | 0,413 | 8,19E-03 | NR_029612 | MIR181B1 | microRNA 181b-1 | NR_029612 |
| **16859655** | 0,413 | 2,16E-03 | NR_000012 | SNORA68 | small nucleolar RNA, H/ACA box 68 | NR_000012 |
| **17020715** | 0,411 | 4,59E-04 | NR_026807 | LINC00472 | long intergenic non-protein coding RNA 472 | NR_026807 |
| **17078190** | 0,408 | 4,36E-03 | NM_007332 | TRPA1 | transient receptor potential cation channel, subfamily A, member 1 | NM_007332 |
| **16918939** | 0,408 | 1,14E-03 | --- | --- | --- | ENST00000561134 |
| **16997399** | 0,407 | 2,52E-03 | NR_003014 | SNORA47 | small nucleolar RNA, H/ACA box 47 | NR_003014 |
| **17063977** | 0,407 | 9,87E-03 | NR_015421 | LOC154761 | family with sequence similarity 115, member C pseudogene | NR_015421 |
| **16798144** | 0,406 | 1,53E-03 | NR_003318 | SNORD116-3 | small nucleolar RNA, C/D box 116-3 | NR_003318 |
| **16798154** | 0,406 | 1,53E-03 | NR_003318 | SNORD116-3 | small nucleolar RNA, C/D box 116-3 | NR_003318 |
| **16798206** | 0,405 | 5,22E-03 | NR_003334 | SNORD116-20 | small nucleolar RNA, C/D box 116-20 | NR_003334 |
| **16798152** | 0,405 | 1,80E-03 | NR_003323 | SNORD116-8 | small nucleolar RNA, C/D box 116-8 | NR_003323 |
| **16984010** | 0,405 | 1,18E-02 | ENST00000303115 | IL7R | interleukin 7 receptor | ENST00000303115 |
| **16788768** | 0,402 | 1,26E-03 | NR_030162 | MIR487A | microRNA 487a | NR_030162 |
| **16967794** | 0,402 | 7,76E-03 | NM_001511 | CXCL1 | chemokine (C-X-C motif) ligand 1 (melanoma growth stimulating activity, alpha) | NM_001511 |
| **16688332** | 0,402 | 3,72E-01 | NM_004675 | DIRAS3 | DIRAS family, GTP-binding RAS-like 3 | NM_004675 |
| **16889530** | 0,401 | 1,17E-02 | ENST00000260967 | CDK15 | cyclin-dependent kinase 15 | ENST00000260967 |
| **17075628** | 0,401 | 9,30E-03 | ENST00000520164 | EBF2 | early B-cell factor 2 | ENST00000520164 |
| **16790362** | 0,401 | 8,31E-04 | NR_002916 | SNORD8 | small nucleolar RNA, C/D box 8 | NR_002916 |
| **16807268** | 0,401 | 1,81E-04 | --- | --- | --- | ENST00000560769 |
| **16870193** | 0,399 | 4,96E-03 | NM_031310 | PLVAP | plasmalemma vesicle associated protein | NM_031310 |
| **16798951** | 0,399 | 4,96E-03 | ENST00000300177 | GREM1 | gremlin 1, DAN family BMP antagonist | ENST00000300177 |
| **17009615** | 0,398 | 1,77E-04 | NR_024403 | LOC730101 | uncharacterized LOC730101 | NR_024403 |
| **16800680** | 0,395 | 8,22E-04 | NM_021199 | SQRDL | sulfide quinone reductase-like (yeast) | NM_021199 |
| **16664003** | 0,395 | 1,81E-03 | NR_000024 | SNORD46 | small nucleolar RNA, C/D box 46 | NR_000024 |
| **17069811** | 0,393 | 1,63E-02 | --- | --- | --- | n370327 |
| **16831224** | 0,392 | 2,15E-03 | NM_001146312 | MYOCD | myocardin | NM_001146312 |
| **16966721** | 0,391 | 6,22E-05 | NR_003016 | SNORA26 | small nucleolar RNA, H/ACA box 26 | NR_003016 |
| **16999421** | 0,391 | 8,46E-03 | NM_178450 | MARCH3 | membrane-associated ring finger (C3HC4) 3, E3 ubiquitin protein ligase | NM_178450 |
| **16705329** | 0,389 | 1,08E-03 | NR_045662 | MYPN | myopalladin | NR_045662 |
| **17050834** | 0,388 | 2,70E-03 | NM_016087 | WNT16 | wingless-type MMTV integration site family, member 16 | NM_016087 |
| **16754134** | 0,388 | 9,99E-02 | NM_003667 | LGR5 | leucine-rich repeat containing G protein-coupled receptor 5 | NM_003667 |
| **17046586** | 0,387 | 5,05E-02 | NR_002961 | SNORA22 | small nucleolar RNA, H/ACA box 22 | NR_002961 |
| **16889268** | 0,383 | 5,34E-04 | ENST00000374700 | AOX1 | aldehyde oxidase 1 | ENST00000374700 |
| **16815310** | 0,383 | 4,96E-03 | NM_016639 | TNFRSF12A | tumor necrosis factor receptor superfamily, member 12A | NM_016639 |
| **16832566** | 0,382 | 2,98E-03 | NR_000014 | SNORD42A | small nucleolar RNA, C/D box 42A | NR_000014 |
| **17045838** | 0,379 | 5,89E-04 | ENST00000275525 | IGFBP1 | insulin-like growth factor binding protein 1 | ENST00000275525 |
| **17080630** | 0,378 | 1,22E-03 | ENST00000395601 | SNTB1 | syntrophin, beta 1 (dystrophin-associated protein A1, 59kDa, basic component 1) | ENST00000395601 |
| **16761326** | 0,377 | 2,18E-02 | NM_001199805 | KLRC4-KLRK1 | KLRC4-KLRK1 readthrough | NM_001199805 |
| **16819794** | 0,376 | 1,45E-02 | NM_001795 | CDH5 | cadherin 5, type 2 (vascular endothelium) | NM_001795 |
| **16950440** | 0,376 | 4,41E-05 | BC137443 | OXTR | oxytocin receptor | BC137443 |
| **17005858** | 0,375 | 1,12E-01 | NM_003509 | HIST1H2AI | histone cluster 1, H2ai | NM_003509 |
| **16788762** | 0,375 | 5,42E-03 | NR_030595 | MIR889 | microRNA 889 | NR_030595 |
| **16844046** | 0,374 | 9,27E-05 | NR_002576 | SNORA21 | small nucleolar RNA, H/ACA box 21 | NR_002576 |
| **16997275** | 0,374 | 2,12E-02 | NM_016591 | GCNT4 | glucosaminyl (N-acetyl) transferase 4, core 2 | NM_016591 |
| **17098491** | 0,373 | 8,22E-04 | AK057602 | RPL12 | ribosomal protein L12 | AK057602 |
| **16788735** | 0,370 | 2,63E-03 | NR_030175 | MIR495 | microRNA 495 | NR_030175 |
| **16798146** | 0,367 | 5,65E-04 | NR_003320 | SNORD116-5 | small nucleolar RNA, C/D box 116-5 | NR_003320 |
| **16798150** | 0,367 | 5,65E-04 | NR_003320 | SNORD116-5 | small nucleolar RNA, C/D box 116-5 | NR_003320 |
| **16798164** | 0,365 | 1,00E-04 | NR_003329 | SNORD116-14 | small nucleolar RNA, C/D box 116-14 | NR_003329 |
| **16768738** | 0,364 | 6,65E-03 | NM_021229 | NTN4 | netrin 4 | NM_021229 |
| **16788770** | 0,363 | 4,24E-03 | NR_029874 | MIR382 | microRNA 382 | NR_029874 |
| **17004903** | 0,360 | 1,25E-03 | NM_001955 | EDN1 | endothelin 1 | NM_001955 |
| **17020995** | 0,359 | 3,33E-04 | ENST00000369947 | HTR1B | 5-hydroxytryptamine (serotonin) receptor 1B, G protein-coupled | ENST00000369947 |
| **16788741** | 0,358 | 2,71E-03 | NR_030390 | MIR654 | microRNA 654 | NR_030390 |
| **16797196** | 0,357 | 5,12E-04 | NM_138420 | AHNAK2 | AHNAK nucleoprotein 2 | NM_138420 |
| **17016403** | 0,356 | 8,33E-02 | NM_003534 | HIST1H3G | histone cluster 1, H3g | NM_003534 |
| **16798148** | 0,355 | 3,01E-04 | NR_003321 | SNORD116-6 | small nucleolar RNA, C/D box 116-6 | NR_003321 |
| **16850216** | 0,353 | 4,53E-02 | NM_003004 | SECTM1 | secreted and transmembrane 1 | NM_003004 |
| **16717520** | 0,353 | 2,38E-03 | NR_002954 | SNORA12 | small nucleolar RNA, H/ACA box 12 | NR_002954 |
| **16798190** | 0,352 | 1,90E-04 | NR_003330 | SNORD116-15 | small nucleolar RNA, C/D box 116-15 | NR_003330 |
| **16788721** | 0,351 | 3,26E-03 | NR_029890 | MIR323A | microRNA 323a | NR_029890 |
| **16785897** | 0,348 | 1,16E-03 | NM_001034852 | SMOC1 | SPARC related modular calcium binding 1 | NM_001034852 |
| **16830912** | 0,347 | 5,85E-04 | NR_037447 | MIR3676 | microRNA 3676 | NR_037447 |
| **16849697** | 0,346 | 2,10E-03 | NM_002522 | NPTX1 | neuronal pentraxin I | NM_002522 |
| **17100667** | 0,346 | 4,01E-02 | --- | --- | --- | NC_001807 |
| **16719125** | 0,342 | 7,49E-02 | NR_037915 | FAM24B-CUZD1 | FAM24B-CUZD1 readthrough | NR_037915 |
| **16798138** | 0,341 | 1,12E-04 | NR_003317 | SNORD116-2 | small nucleolar RNA, C/D box 116-2 | NR_003317 |
| **16716718** | 0,339 | 1,17E-02 | NR_033969 | PLCE1-AS1 | PLCE1 antisense RNA 1 | NR_033969 |
| **16761350** | 0,336 | 1,08E-02 | NM_002261 | KLRC3 | killer cell lectin-like receptor subfamily C, member 3 | NM_002261 |
| **16885842** | 0,331 | 2,46E-04 | NM_001508 | GPR39 | G protein-coupled receptor 39 | NM_001508 |
| **16923200** | 0,327 | 2,70E-03 | NM_018964 | SLC37A1 | solute carrier family 37 (glycerol-3-phosphate transporter), member 1 | NM_018964 |
| **16914743** | 0,324 | 2,32E-04 | NR_003695 | SNORD12B | small nucleolar RNA, C/D box 12B | NR_003695 |
| **17051553** | 0,323 | 4,19E-03 | NM_016352 | CPA4 | carboxypeptidase A4 | NM_016352 |
| **17005865** | 0,319 | 6,52E-02 | NM_003521 | HIST1H2BM | histone cluster 1, H2bm | NM_003521 |
| **16907324** | 0,318 | 8,72E-04 | ENST00000409474 | MPP4 | membrane protein, palmitoylated 4 (MAGUK p55 subfamily member 4) | ENST00000409474 |
| **16952782** | 0,317 | 2,36E-04 | NM_015444 | TMEM158 | transmembrane protein 158 (gene/pseudogene) | NM_015444 |
| **16990203** | 0,315 | 7,43E-03 | NR_026705 | VTRNA1-3 | vault RNA 1-3 | NR_026705 |
| **17073220** | 0,315 | 4,08E-04 | NM_001160354 | LY6K | lymphocyte antigen 6 complex, locus K | NM_001160354 |
| **16697938** | 0,315 | 1,42E-04 | NM_001193571 | CSRP1 | cysteine and glycine-rich protein 1 | NM_001193571 |
| **16963113** | 0,315 | 4,28E-03 | NM_001647 | APOD | apolipoprotein D | NM_001647 |
| **17091502** | 0,311 | 4,28E-02 | ENST00000371625 | PTGDS | prostaglandin D2 synthase 21kDa (brain) | ENST00000371625 |
| **16909524** | 0,307 | 1,97E-02 | ENST00000365530 | SNORD82 | small nucleolar RNA, C/D box 82 | ENST00000365530 |
| **16837938** | 0,306 | 1,08E-03 | NM_001005619 | ITGB4 | integrin, beta 4 | NM_001005619 |
| **16837063** | 0,301 | 9,32E-03 | NR_002995 | SNORA76 | small nucleolar RNA, H/ACA box 76 | NR_002995 |
| **17019698** | 0,290 | 7,65E-03 | NM_001251973 | RCAN2 | regulator of calcineurin 2 | NM_001251973 |
| **16788749** | 0,289 | 4,09E-03 | NR_031575 | MIR1185-1 | microRNA 1185-1 | NR_031575 |
| **16744317** | 0,284 | 1,62E-02 | OTTHUMT00000391479 // OTTHUMT00000391479 | OTTHUMG00000166794 // RP11-108O10.2 | NULL // NULL | OTTHUMT00000391479 |
| **16948561** | 0,275 | 2,63E-03 | --- | --- | --- | ENST00000364359 |
| **16788743** | 0,268 | 6,22E-05 | NR_030157 | MIR376B | microRNA 376b | NR_030157 |
| **16937645** | 0,267 | 7,35E-03 | NM_003042 | SLC6A1 | solute carrier family 6 (neurotransmitter transporter, GABA), member 1 | NM_003042 |
| **16915091** | 0,264 | 1,13E-04 | NM_003222 | TFAP2C | transcription factor AP-2 gamma (activating enhancer binding protein 2 gamma) | NM_003222 |
| **16798216** | 0,243 | 3,41E-04 | NR_003338 | SNORD116-24 | small nucleolar RNA, C/D box 116-24 | NR_003338 |
| **16743721** | 0,237 | 8,31E-04 | NM_002421 | MMP1 | matrix metallopeptidase 1 (interstitial collagenase) | NM_002421 |
| **16754402** | 0,224 | 6,22E-05 | --- | --- | --- | ENST00000553247 |
| **17025564** | 0,221 | 1,00E-03 | NR_026860 | LINC00473 | long intergenic non-protein coding RNA 473 | NR_026860 |
| **16788739** | 0,218 | 9,26E-03 | NR_030266 | MIR376A2 | microRNA 376a-2 | NR_030266 |
| **17090296** | 0,203 | 1,86E-04 | NM_000050 | ASS1 | argininosuccinate synthase 1 | NM_000050 |
| **16774303** | 0,192 | 2,03E-06 | NM_014059 | RGCC | regulator of cell cycle | NM_014059 |
| **16986433** | 0,134 | 6,22E-05 | NM_001882 | CRHBP | corticotropin releasing hormone binding protein | NM_001882 |
| **17090320** | 0,044 | 2,13E-07 | --- | --- | --- | ENST00000458976 |
