# Supplementary material for: TGF‐β‐induced IGFBP‐3 is a key paracrine factor from activated pericytes that promotes colorectal cancer cell migration and invasion
Source: Mol Oncol. 2020 Sep 1;14(10):2609–28. doi: 10.1002/1878-0261.12779 (PMC7530788; doi:10.1002/1878-0261.12779)
Supplement: Supplementary file 8 — Table S3. Gene ontology analysis of genes significantly upregulated (fold change >2, FDR < 0.05) from Table S2. [file MOL2-14-2609-s008.docx]

**Supplementary Table S3 –** Gene Ontology analysis of genes significantly upregulated in human primary pericytes cocultured with CRC cells

| **Category** | **Term** | **P-Value** | **Fold Enrichment** | **Bonferroni** | **Benjamini** | **FDR** |
| --- | --- | --- | --- | --- | --- | --- |
| GOTERM_BP_FAT | GO:0048514~blood vessel morphogenesis | 1,74E-10 | 9,16 | 2,60E-07 | 2,60E-07 | 2,89E-07 |
| GOTERM_BP_FAT | GO:0001568~blood vessel development | 1,40E-09 | 7,89 | 2,09E-06 | 1,04E-06 | 2,32E-06 |
| GOTERM_BP_FAT | GO:0001944~vasculature development | 1,95E-09 | 7,70 | 2,91E-06 | 9,71E-07 | 3,24E-06 |
| GOTERM_BP_FAT | GO:0001525~angiogenesis | 3,63E-07 | 8,98 | 5,43E-04 | 1,36E-04 | 6,03E-04 |
| GOTERM_BP_FAT | GO:0030334~regulation of cell migration | 1,23E-06 | 7,86 | 1,84E-03 | 3,69E-04 | 2,05E-03 |
| GOTERM_BP_FAT | GO:0042127~regulation of cell proliferation | 1,40E-06 | 3,38 | 2,09E-03 | 3,49E-04 | 2,33E-03 |
| GOTERM_BP_FAT | GO:0040012~regulation of locomotion | 3,91E-06 | 6,92 | 5,82E-03 | 8,33E-04 | 6,49E-03 |
| GOTERM_BP_FAT | GO:0051270~regulation of cell motion | 4,09E-06 | 6,88 | 6,09E-03 | 7,64E-04 | 6,79E-03 |
| GOTERM_BP_FAT | GO:0051173~positive regulation of nitrogen compound metabolic process | 4,66E-06 | 3,56 | 6,94E-03 | 7,74E-04 | 7,74E-03 |
| GOTERM_BP_FAT | GO:0008284~positive regulation of cell proliferation | 7,14E-06 | 4,38 | 1,06E-02 | 1,07E-03 | 1,19E-02 |
| GOTERM_BP_FAT | GO:0007179~transforming growth factor beta receptor signaling pathway | 9,30E-06 | 14,09 | 1,38E-02 | 1,26E-03 | 1,54E-02 |
| GOTERM_BP_FAT | GO:0031328~positive regulation of cellular biosynthetic process | 1,09E-05 | 3,35 | 1,61E-02 | 1,36E-03 | 1,81E-02 |
| GOTERM_MF_FAT | GO:0003700~transcription factor activity | 1,19E-05 | 2,84 | 3,37E-03 | 3,37E-03 | 1,58E-02 |
| GOTERM_BP_FAT | GO:0045935~positive regulation of nucleic acid metabolic process | 1,24E-05 | 3,48 | 1,83E-02 | 1,42E-03 | 2,05E-02 |
| GOTERM_BP_FAT | GO:0035295~tube development | 1,30E-05 | 6,04 | 1,92E-02 | 1,38E-03 | 2,16E-02 |
| GOTERM_BP_FAT | GO:0009891~positive regulation of biosynthetic process | 1,33E-05 | 3,30 | 1,96E-02 | 1,32E-03 | 2,20E-02 |
| GOTERM_BP_FAT | GO:0007178~receptor protein serine/threonine kinase signaling pathway | 2,15E-05 | 9,38 | 3,15E-02 | 2,00E-03 | 3,56E-02 |
| GOTERM_BP_FAT | GO:0007166~cell surface receptor linked signal transduction | 2,35E-05 | 2,15 | 3,45E-02 | 2,06E-03 | 3,90E-02 |
| GOTERM_MF_FAT | GO:0046332~SMAD binding | 3,63E-05 | 15,68 | 1,02E-02 | 5,14E-03 | 4,80E-02 |

BP: Biological Process; MF, Molecular Function
