# Supplementary material for: TGF‐β‐induced IGFBP‐3 is a key paracrine factor from activated pericytes that promotes colorectal cancer cell migration and invasion
Source: Mol Oncol. 2020 Sep 1;14(10):2609–28. doi: 10.1002/1878-0261.12779 (PMC7530788; doi:10.1002/1878-0261.12779)
Supplement: Supplementary file 9 — Table S4. GSEA analysis of gene sets significantly enriched (FDR < 0.05) in human primary pericytes cocultured with CRC cells. [file MOL2-14-2609-s009.docx]

**Supplementary Table S4.** GSEA (gene set enrichment analysis) of gene sets significantly upregulated (FDR < 0.05) in human primary pericytes cocultured with CRC cells.

| **Hallmarks** | **Size** | **ES** | **NES** | **NOM p-val** | **FDR q-val** |
| --- | --- | --- | --- | --- | --- |
| TGF-β signaling | 53 | 0,6324248 | 2,3436456 | <1E-04 | <1E-04 |
| UV response | 140 | 0,4329783 | 1,9003546 | <1E-04 | 8,38E-04 |
| TNF-α signaling via NF-kB | 198 | 0,4073781 | 1,8742914 | <1E-04 | 6,57E-04 |
| NOTCH signaling | 31 | 0,5471198 | 1,8005178 | 0,00325052 | 0,00192359 |
| WNT/β-catenin signaling | 41 | 0,4832874 | 1,6958259 | 0,00625 | 0,00524364 |
| Epithelial-mesenchymal transition | 198 | 0,35939473 | 1,6618031 | <1E-04 | 0,00605681 |
| HEDGEHOG signaling | 35 | 0,4869568 | 1,6527932 | 0,01098901 | 0,00572806 |
| Angiogenesis | 36 | 0,482388 | 1,6460814 | 0,01037247 | 0,00535148 |
| Apoptosis | 159 | 0,3301937 | 1,4798764 | 0,00479489 | 0,02247288 |
| Hypoxia | 197 | 0,31375352 | 1,4400487 | 0,00583554 | 0,0289693 |
